# Deconvolution of subcellular protrusion heterogeneity and the underlying actin regulator dynamics from live cell imaging

**DOI:** 10.1038/s41467-018-04030-0

**Published:** 2018-04-27

**Authors:** Chuangqi Wang, Hee June Choi, Sung-Jin Kim, Aesha Desai, Namgyu Lee, Dohoon Kim, Yongho Bae, Kwonmoo Lee

**Affiliations:** 1 Department of Biomedical Engineering, Worcester Polytechnic Institute, Worcester, MA 01609 USA; 2 Department of Pathology and Anatomical Sciences, Computational Cell Biology, Anatomy and Pathology Program, Jacobs School of Medicine and Biomedical Sciences, University at Buffalo, State University of New York, Buffalo, NY 14203 USA; 3 Department of Molecular, Cell and Cancer Biology, University of Massachusetts Medical School, Worcester, MA 01655 USA

## Abstract

Cell protrusion is morphodynamically heterogeneous at the subcellular level. However, the mechanism of cell protrusion has been understood based on the ensemble average of actin regulator dynamics. Here, we establish a computational framework called HACKS (deconvolution of heterogeneous activity in coordination of cytoskeleton at the subcellular level) to deconvolve the subcellular heterogeneity of lamellipodial protrusion from live cell imaging. HACKS identifies distinct subcellular protrusion phenotypes based on machine-learning algorithms and reveals their underlying actin regulator dynamics at the leading edge. Using our method, we discover “accelerating protrusion”, which is driven by the temporally ordered coordination of Arp2/3 and VASP activities. We validate our finding by pharmacological perturbations and further identify the fine regulation of Arp2/3 and VASP recruitment associated with accelerating protrusion. Our study suggests HACKS can identify specific subcellular protrusion phenotypes susceptible to pharmacological perturbation and reveal how actin regulator dynamics are changed by the perturbation.

## Abstract (web summary)

Cell protrusion dynamics are heterogeneous at the subcellular level, but current analyses operate at the cellular or ensemble level. Here the authors develop a computational framework to quantify subcellular protrusion phenotypes and reveal the underlying actin regulator dynamics at the leading edge.

## Introduction

Cell protrusion is driven by spatiotemporally fluctuating actin assembly processes, and is morphodynamically heterogeneous at the subcellular level^1–3^. Elucidating the underlying molecular dynamics associated with subcellular protrusion heterogeneity is crucial to understanding the biology of cellular movement since protrusion determines the directionality and persistence of cell movements or facilitates the exploration of the surrounding environment^4^. Recent studies of the vital roles of cell protrusion in tissue regeneration^5,6^, cancer invasiveness and metastasis^7–9^, and the environmental exploration of leukocytes^10^ further emphasize the physiological and pathophysiological implication of understanding the fine molecular details of protrusion mechanisms. Although there has been considerable progress in analyzing individual functions of actin regulators, the precise understanding of how these actin regulators are spatiotemporally acting in cell protrusion is still limited. Moreover, it is a formidable task to dissect the actin regulator dynamics involved with cell protrusion because such dynamics are highly heterogeneous and fluctuate on both the micron length scale and the minute time scale^11–13^.

Advances in computational image analysis on live cell movies have allowed us to study the dynamic aspects of molecular and cellular events at the subcellular level. However, the significant degree of heterogeneity in molecular and subcellular dynamics complicates the extraction of useful information from complex cellular behavior. The current method of characterizing molecular dynamics involves averaging molecular activities at the cellular level, which significantly conceals the fine differential subcellular coordination of dynamics among actin regulators. Over the past decade, hidden variable cellular phenotypes in heterogeneous cell populations have been uncovered by applying machine learning analyses^14,15^; however, these analyses primarily focused on static data sets acquired at the single-cell level, such as immunofluorescence^16^, mass cytometry^17^, and single-cell RNA-Seq^18^ data sets. Although some studies have examined the cellular heterogeneity of the migratory mode^19,20^, subcellular protrusion heterogeneity has not yet been addressed. Moreover, elucidating the molecular mechanisms that generate each subcellular phenotype has been experimentally limited because it is a challenging task to manipulate specific subclasses of molecules at the subcellular level with fine spatiotemporal resolution.

**Fig. 1:**
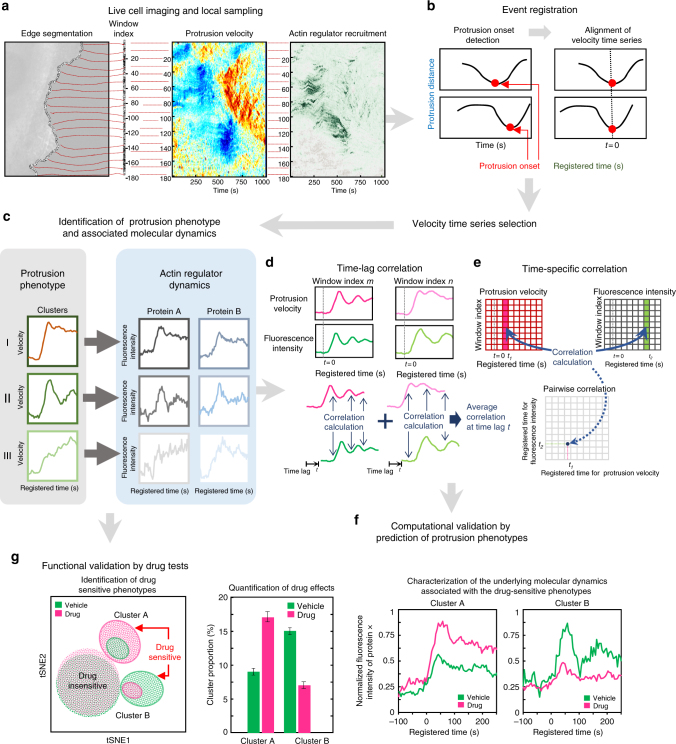
Schematic representation of the analytical steps of HACKS. **a** Fluorescence time-lapse movies of the leading edge of a migrating PtK1 cell expressing flourescent-tagged proteins of interest (an Arp3-HaloTag expressing cell is presented here) was taken at 5 s per frame, and then probing windows (500 by 500 nm) are generated to track the cell edge movement and sample protrusion velocities and fluorescence intensities. **b** The protrusion distance is registered with respect to protrusion onsets (*t* = 0). Time series of protrusion velocities are then aligned. **c** The protrusion phenotypes are identified by a time series clustering analysis and associated with actin regulator dynamics. **d**–**f** Correlation analysis between time series of the protrusion velocities and fluorescence intensities. Schematic diagrams of time-lag (**d**) and time-specific correlation analysis (**e**) are presented. Classification analysis is performed to computationally validate the result by predicting protrusion phenotypes based on molecular dynamics. **g** The hypotheses drawn from the computational analysis are functionally validated by drug tests. The phenotypes susceptible to pharmacological perturbations are identified based on t-SNE plots. The drug-sensitive phenotypes are further analyzed by quantifying the drug effects on cluster proportion and the associated molecular dynamics

To address this challenge, we developed a machine learning-based computational analysis pipeline that we have called HACKS (deconvolution of Heterogeneous Activity in Coordination of cytosKeleton at the Subcellular level) (Fig. 1) for live cell imaging data by an unsupervised machine learning approach combined with our local sampling and registration method^13^. HACKS allows us to deconvolve the subcellular heterogeneity of protrusion phenotypes and statistically link them to the dynamics of actin regulators at the leading edge of migrating cells. Based on our method, we quantitatively identify subcellular protrusion phenotypes from highly heterogeneous and non-stationary edge dynamics of migrating epithelial cells. Each protrusion phenotype is demonstrated to be associated with the differential temporal coordination of the actin regulators at the leading edge. Analyzing pharmacologically perturbed cells further verifies that the fine temporal coordination of the actin regulators is required to generate specific subcellular protrusion phenotypes.

## Results

### HACKS: an overview of the pipeline

To deconvolve the heterogeneity of the subcellular protrusion activity and their regulatory proteins at fine spatiotemporal resolution, we developed a computational analysis pipeline, HACKS (Fig. 1), which is based on an unsupervised machine learning method. HACKS allowed us to (i) identify distinct subcellular protrusion phenotypes based on a time series clustering analysis of heterogeneous subcellular protrusion velocities extracted from live cell movies (Figs. 1a–c), (ii) associate each protrusion phenotype with pertinent actin regulator dynamics by comparing the average temporal patterns of protrusion velocities with those of actin regulators (Fig. 1c), (iii) perform highly specified correlation and classification analyses of actin regulator dynamics of protrusion phenotypes to establish their association with fine mechanistic details (Figs. 1d–f) and (iv) identify specific protrusion phenotypes susceptible to molecular perturbations, and functionally confirm the association between protrusion phenotype and the actin regulator dynamics (Fig. 1g). The framework can provide mechanistic insight into how the differential coordination of actin regulator dynamics organizes various subcellular protrusion phenotypes.

**Fig. 2:**
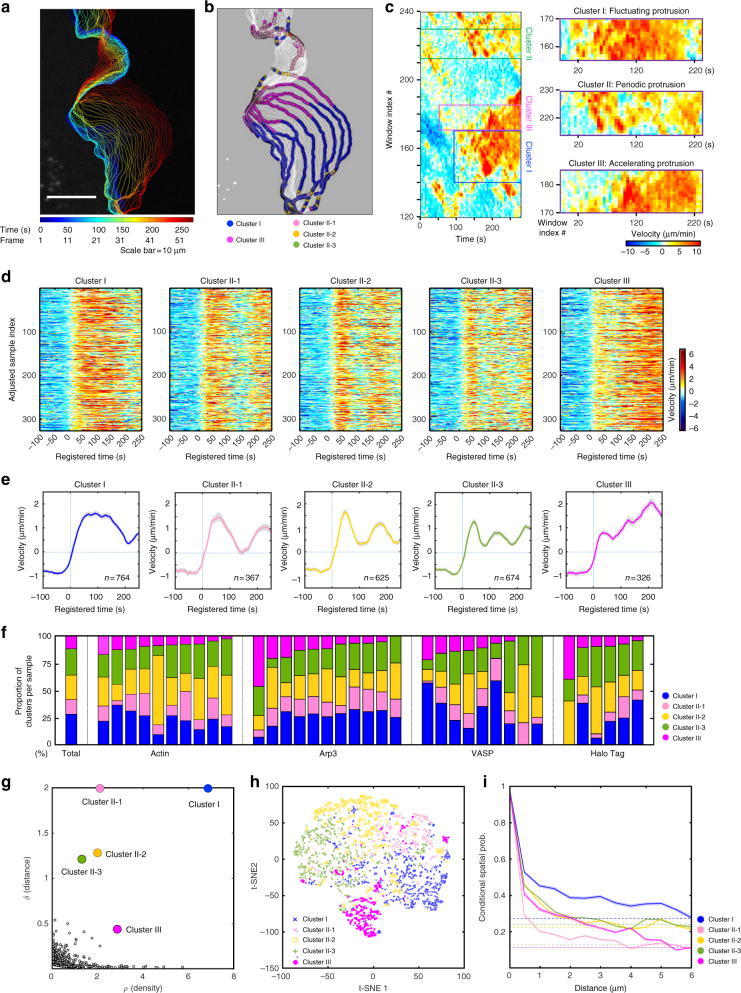
Subcellular protrusion phenotypes revealed by a time series clustering analysis. **a**–**c** A representative cluster assignment on a time-lapse movie of a PtK1 cell stained with CellMask DeepRed. Edge evolution on 5 s interval (**a**), cluster assignments of each probing window on every four frame (20 s interval) (**b**), and the space-time maps of instantaneous edge velocity (**c**) of the entire cell edge and the indicated cluster regions. Scale bar: 10 μm. **d** Raw velocity maps for Cluster I, II-1, II-2, II-3, and III. All time series are registered with respect to protrusion onset (*t* = 0). **e** Average time series of protrusion velocity registered at protrusion onsets (*t* = 0) in each cluster. Solid lines indicate population averages. Shaded error bands indicate 95% confidence intervals of the mean computed by bootstrap sampling. *n* indicates the number of time series in each cluster. The time lapse movies of 36 cells were used in this analysis. **f** Proportions of each cluster in entire samples or individual cells expressing fluorescent actin, Arp3, VASP, and HaloTag, respectively. **g** Decision graph of the density peak clustering analysis of protrusion velocities. **h** A t-SNE plot of the autocorrelation functions of protrusion velocity time series overlaid with cluster assignments. **i** Spatial conditional distribution of each cluster. Solid lines indicate population averages. Shaded error bands indicate 95% confidence intervals of the mean computed by bootstrap sampling

**Fig. 3:**
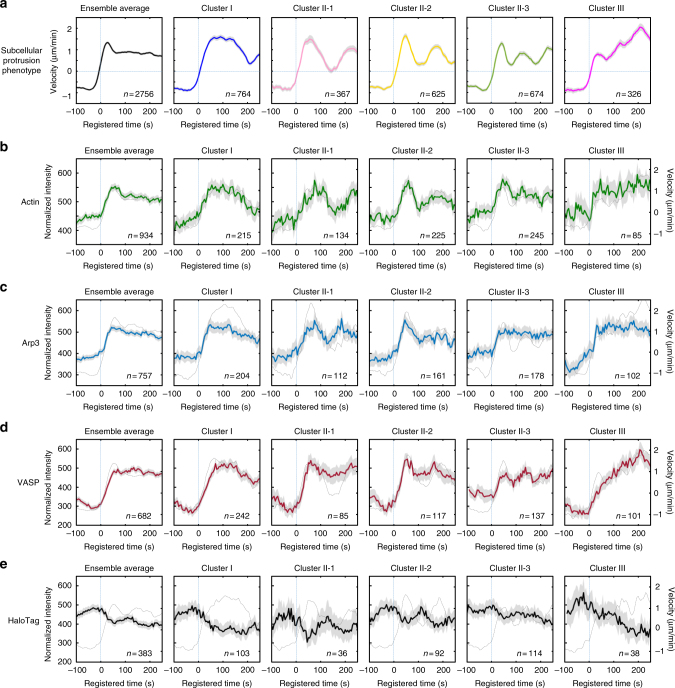
Distinctive actin regulator dynamics associated with subcellular protrusion phenotypes. **a** Ensemble averaged velocity time series of entire samples and averaged velocity time series sampled in each cluster. All time series are registered with respect to protrusion onset (*t* = 0). **b**–**e** Ensemble averaged normalized fluorescence intensity time series of entire samples and normalized fluorescence intensity time series in each cluster. All time series are registered with respect to protrusion onset (*t* = 0). Solid lines indicate population averages. Shaded error bands indicate 95% confidence intervals of the mean computed by bootstrap sampling. The dotted lines in **b**-**e** indicate protrusion velocity time series associated with the indicated fluorescent proteins. *n* indicates the number of time series sampled in each cluster. The numbers of cells used for the analyses are 36 (**a**), 10 (**b**), 11 (**c**), 9 (**d**) and 6 (**e**) respectively. The number of time series sampled and the number of cells imaged for each cluster is summarized in Supplementary Table 1

**Fig. 4:**
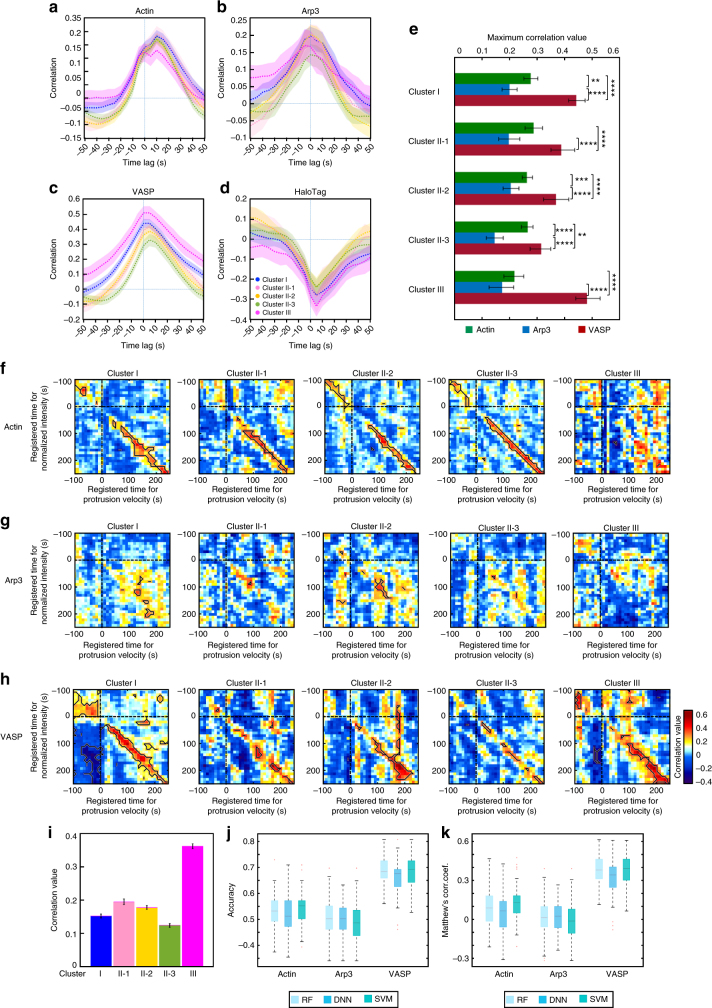
Correlation and classification analyses between protrusion velocity and actin regulator dynamics. **a**–**d** Time-lag correlation analysis based on Pearson’s cross-correlation of edge velocity and actin (**a**), Arp3 (**b**), VASP (**c**), and HaloTag (**d**). Solid lines indicate population averages. Shaded error bands indicate 95% confidence intervals of the mean computed by bootstrap sampling. The number of samples used for the analysis is identical from Fig.3b–e. **e** Comparison and statistical testing of maximum correlation coefficients from **a**–**d** in each cluster. The error bar indicates 95% confidence interval of the mean by bootstrapping. **p < 0.01, ***p < 0.001 and ****p < 0.0001 indicate the statistical significance by two-tailed two-sample Kolmogorov–Smirnov (KS) test. The *p*-values are listed in Supplementary Table 3. **f**–**h** Time-specific correlation analysis based on pairwise Pearson’s correlation coefficients of protrusion velocity and fluorescence intensity time series registered relative to protrusion onset. The regions surrounded by the black lines are statistically significant correlation by Benjamini-Hochberg multiple hypothesis testing. **i** Pearson’s correlation coefficients between early Arp3 intensities and late protrusion velocities in each cluster. The error bar indicates 95% confidence interval of the mean by bootstrapping. The numbers of samples in this analysis are 204 (Cluster I), 112 (Cluster II-1), 161 (Cluster II-2), 178 (Cluster II-3) and 102 (Cluster III) respectively. **j**–**k** Classification analysis of Cluster III against Clusters I/II based on fluorescent intensity time series. Boxplots of the accuracy (**j**) and Matthews correlation coefficients (**k**) represent multiple classification results. RF stands for Random Forest, DNN for Deep Neural Network, and SVM for Support Vector Machine. The central line indicates median, and both edges of the box each represent 25^th^ and 75^th^ percentiles. The numbers of samples used in these analyses are 934 (actin), 757 (Arp3) and 682 (VASP) respectively

### A time series clustering analysis of protrusion velocities

Sample videos for the analysis were prepared by taking time-lapse movies of PtK1 epithelial cells expressing fluorescently tagged actin, Arp3, VASP and a cytoplasmic marker, HaloTag, with a spinning disk confocal microscope for approximately 200 frames at 5 s per frame^11^ (Fig. 1a). Each time-lapse movie contains a single cell whose leading edge undergoes protrusion–retraction cycles. After segmenting the leading edge of each cell by multiple probing windows with a size of 500 by 500 nm^13^ (Fig. 1a, left), time series of velocities^11^ and fluorescence intensities of the tagged molecules^12,13^ acquired from each probing window were quantified (Fig. 1a, center and right). After registering protrusion onset at time zero (*t* = 0), the time-series were aligned using the protrusion onset as a temporal fiduciary^13^ (Fig. 1b). To ensure a uniform time length of the data for the subsequent clustering analysis, we selected the first 51 frames (250 s) of protrusion segments, which is about the average protrusion duration^13^ from the pooled velocity time series.

The selected time series of the registered protrusion velocity contained a substantial amount of intrinsic fluctuations, hindering the identification of distinct clusters of similar protrusion activities. Therefore, we first denoised the time series velocity profile using empirical mode decomposition (EMD)^21^ and discretized the data using SAX (symbolic aggregate approximation, see Methods)^22^ to reduce the dimensionality and complexity of the data (Supplementary Note 1). We then extracted distinct patterns from fluctuating velocity time series by combining the autocorrelation distance measure with the density peak clustering^23^. The distance measures between different time series were calculated using the squared Euclidean distances between the corresponding autocorrelation functions (ACFs) of each discretized time series. This autocorrelation distance partitioned the fluctuating time series of similar underlying patterns into the same clusters, enabling us to identify clusters with distinct dynamic patterns (Supplementary Note 1). Following the ACF distance measure, we applied the density peak clustering algorithm, which has been shown to be superior to conventional *k*-means in partitioning data with complex cluster shapes^23^. As a result, the density–distance graph in Fig. 2g, where cluster centers are localized in the upper-right region (see Methods for detail), revealed five distinct clusters of subcellular protrusion activities. Using the clustering criteria, Davies–Bouldin Index (DBI)^24^, average silhouette value, and Calinski–Harabasz pseudo *F*-statistic^25^, we also confirmed that the optimal number of clusters was five (Supplementary Fig. 3a–c). After the clustering analysis, average protrusion velocities and the 95% confidence intervals of the mean were calculated (Fig. 2e). Of note, after we tested different sets of algorithms, we found that ACF distance was the most important factor which allowed us to extract these distinct temporal patterns (Supplementary Figs. 1 and 2, Supplementary Note 1). Furthermore, we could not identify substantial differences among the velocity cluster profiles (dotted lines in Fig. 3b–e) in each molecule (actin, Arp3, VASP, and HaloTag), confirming that our clustering results are not skewed by a particular data set. The numbers of cells and probed windows used in the time series clustering analysis are presented in Supplementary Table 1.

### Identification of distinct subcellular protrusion phenotypes

The visual inspection of the average velocity profiles of the identified clusters (Fig. 2e) demonstrated that the overall differences among the protrusion phenotypes originated from differences in the timing and number of peaks the velocity reached. Whereas Cluster I did not exhibit dramatic changes in protrusion velocities after reaching its peak at the earlier part of the protrusion segment (Fig. 2e), the remaining clusters exhibited substantial acceleration or deceleration in the protrusion velocities with varying timing and number. Clusters II-1, II-2, and II-3 (Fig. 2e) exhibited differential periodic changes in the acceleration and deceleration of protrusion. Conversely, Cluster III (Fig. 2e) demonstrated persistently accelerating behavior where protrusion velocities continued to increase until the late phase of the protrusion. Clusters I, II-1, II-2, II-3 and III comprised 27.7%, 13.3%, 22.7%, 24.5%, and 11.8% of the entire sample, respectively, and individual cells expressing different fluorescent proteins exhibited similar tendencies (Fig. 2f, Supplementary Fig. 3h), suggesting the intracellular origin of protrusion heterogeneity. Nevertheless, cell-to-cell variability in cluster distribution persisted, suggesting that the clusters may also reflect individual cellular responses to differential cellular contexts or microenvironments.

The validity of our clustering result was confirmed by visually inspecting the velocity activity map (Fig. 2d). Clusters II-1/2/3 (Fig. 2d) and III (Fig. 2d) exhibited clearly distinguishable patterns, whereas Cluster I (Fig. 2d) contained fluctuating velocity profiles (See Supplementary Fig. 3g for the full maps). The t-SNE (Fig. 2h), multidimensional scaling (MDS), silhouette, and order distance plots (Supplementary Fig. 3d–f) of the clustering results further confirmed the stability and tightness of Clusters II-1/2/3 and III but suggested residual heterogeneity in Cluster I, which is in agreement with the velocity activity maps (Fig. 2d). To quantify the spatial structure of the protrusion phenotypic clusters, we estimated the conditional probability that the same cluster exists over the distance from a given cluster (Fig. 2i). As the distance increases between two neighboring clusters, this conditional probability in all clusters decreases to their basal levels of the cluster proportions (Fig. 2i). The conditional probability in Cluster II-1/2/3 quickly decreased within 2 μm distance whereas those in Cluster I and III persisted up to 5 μm (Fig. 2i). These data suggest that Clusters I and III aggregate and act more collectively compared to Cluster II-1/2/3. In addition to PtK1 cells, we further performed the same analysis on MCF10A, human mammary epithelial cells. MCF10A also had very similar subcellular protrusion phenotypes (Supplementary Fig. 4), suggesting that the identified subcellular protrusion phenotypes by HACKS are not limited to a specific cell line.

The visualization of the edge evolution (Fig. 2a), the cluster assignments evolution (Fig. 2b), and the protrusion velocity map (Fig. 2c) of the exemplified live cell movie representatively manifested the morphodynamic features of each subcellular protrusion phenotype (Supplementary Movie 1). Based on our observation, Cluster I is named “fluctuating protrusion” because of the irregularity of its velocity profiles. Since Cluster II-1/2/3 clearly exhibit periodic edge evolution, we refer to Cluster II-1/2/3 collectively as “periodic protrusion”. Notably, Cluster III shows accelerating edge evolution, and, therefore, we refer to Cluster III as “accelerating protrusion”.

### Differential molecular dynamics of actin regulators

We hypothesized that the distinctive subcellular protrusion phenotypes arise from the differential spatiotemporal regulation of actin regulators. Therefore, we next investigated the relationship between the velocity profiles of each protrusion phenotype and the fluctuation of the signal intensities of actin and several actin regulators for each protrusion phenotype. We selected a set of fluorescently tagged molecules to be expressed and monitored; SNAP-tag-actin, HaloTag-Arp3 (tagged on the C-terminus), which represented the Arp2/3 complex involved in actin nucleation, and HaloTag-VASP or GFP-VASP, which represented actin elongation. A diffuse fluorescent marker, HaloTag labeled with tetramethylrhodamine (TMR) ligands^26^, was used as a control signal. The fluorescence intensities of each tagged molecule were acquired from each probing window along with the protrusion velocities (Fig. 1a). The time-series of the fluorescence intensities of each molecule were then grouped and averaged according to the assigned protrusion phenotype (Figs. 1c and 3b–e).

Whereas the molecular dynamics of actin, Arp3 and VASP all exhibited patterns similar to those of the velocity profiles in Clusters I and II-1 (Fig. 3b–d, Cluster I/II-1 each), the Arp3 temporal patterns became less correlated with those of protrusion velocity in Cluster II-2 and II-3 as the frequency of the oscillation increased (Fig. 3c, Cluster II-1/2/3). This demonstrates that underlying molecular temporal patterns can be highly variable depending on the dynamic properties of protrusion activities. Intriguingly, Cluster III also exhibited distinctive molecular dynamics in relation to velocity profiles (Fig. 3a–d, Cluster III each). Whereas the protrusion velocity continued to increase until the late stages of the protrusion segment in the accelerating protrusion (Fig. 3a, Cluster III), the actin fluorescence intensity soon reached its maximum in the early phase and remained constant (Fig. 3b, Cluster III). This pattern indicates that edge movement during accelerating protrusion is driven by the elongation of existing actin filaments rather than de novo actin nucleation. Conversely, Clusters I and II-1/2/3 exhibited increased actin intensity at the leading edge along with increased protrusion velocity (Fig. 3b, Cluster I, II-1/2/3), indicating that actin nucleation mediates subcellular protrusion.

In accordance with the plateaued actin intensities in Cluster III (Fig. 3b, Cluster III), the Arp3 intensity remained constant after reaching its peak in the early protrusion phase (Fig. 3c, Cluster III), whereas the VASP intensities began to increase at protrusion onset and continued to increase (Fig. 3d, Cluster III). These findings suggest that actin elongation by VASP plays a crucial role in driving accelerating protrusion. Whereas the Arp2/3 complex has been considered as a major actin nucleator that drives lamellipodial protrusion^27^, the Arp2/3 complex seemed to play a role in the earlier part of the protrusion in accelerating protrusion. Approximately 50 s after protrusion onset, the Arp3 intensity reached its peak (Fig. 3c, Cluster III), and the acceleration temporarily stopped (Fig. 3a, Cluster III). Notably, the Arp3 intensities began to increase approximately 50 s prior to the protrusion onset in Cluster III (Fig. 3c, Cluster III), whereas they began to increase at the onset of the protrusion in Clusters I and II-1/2/3 (Fig. 3c, Cluster I, II-1/2/3). These findings imply that there exists specific temporal coordination where the Arp2/3 complex nucleates actin networks in the early phase, and VASP then elongates actin filaments to drive the later stages of accelerating protrusion. The specificity of the relationship between the protrusion phenotypes and the underlying molecular dynamics was further validated with a control experiment using HaloTag-TMR (Fig. 3e). Diffused cytoplasmic fluorescence did not exhibit any cluster-specific pattern. Instead, it inversely correlated with the protrusion velocity, suggesting that the cell edges become thinner as the protrusion velocity increases^13^. Notably, the differential dynamics of Arp3 and VASP were not observed when the entire time series data set was ensemble averaged^13^ (Fig. 3c, d, ensemble average each). These results demonstrate the power of our computational framework in revealing the hidden differential subcellular dynamics of actin regulators involved in the generation of heterogeneous morphodynamic phenotypes.

### VASP recruitment correlates with protrusion velocity

To quantitatively assess the coordination between protrusion velocities and the dynamics of actin regulators, we performed a time-lag correlation analysis by calculating Pearson’s correlation coefficients between protrusion velocities and actin regulator intensities with varying time lags in the same windows and averaged over different sampling windows (Fig. 1d). For actin and Arp3, the significant but relatively weak correlations were identified between the protrusion velocities and the intensities in all clusters (Fig. 4a, b). Conversely, the correlation of VASP in all clusters was stronger, particularly the correlation in Cluster III being the strongest in all clusters (Fig. 4c). Consistent with the results of cytoplasmic dynamics (Fig. 3e), HaloTag-TMR intensities were negatively correlated with protrusion velocities (Fig. 4d). Furthermore, a comparison of the maximum correlations in each cluster showed that VASP exhibited significantly stronger correlations than the Arp2/3 complex in all clusters (Fig. 4e, *p*-values in Supplementary Table 3, two-tailed Kolmogorov–Smirnov (K–S) test). These findings suggest that VASP may play a more direct role in mediating protrusion velocities in all clusters than Arp2/3.

Although the above-described conventional time correlation analysis effectively demonstrated the overall correlation between molecular dynamics and the protrusion velocity, its ability to reveal changes in this correlation over time as the protrusion progresses is limited. In other words, the correlation between the protrusion velocities and the fluorescence intensities for each specific time point was not examined in the previous analyses (Fig. 4a–d). Therefore, we performed sample-based correlation analyses whereby calculating pairwise Pearson correlation coefficients, ${\mathrm{c}}(\left\{ V \right\}_{t_i},\left\{ I \right\}_{t_j})$, between the sample of the protrusion velocity, $\left\{ V \right\}_{t_i}$, at the registered time, *t*_*i*_, and the sample of the actin regulator intensity $\left\{ I \right\}_{t_j}$, at the registered time,*t*_*j*_, over the entire probing window population (Fig. 1e)^13^. Then, the statistical significance of the correlations were tested by Benjamini–Hochberg multiple testing^28^.

As expected, the pairwise time correlation analysis between the actin intensities and protrusion velocities (Fig. 4f) further supported the proposition that accelerating protrusions are mediated by the elongation of pre-existing actin filaments, whereas actin nucleation is responsible for non-accelerating protrusions. The significant regions (the black boundaries in Fig. 4f) of instantaneous positive correlations between the actin intensities and protrusion velocities at the leading edge found in Clusters I and II-1/2/3 (Fig. 4f, Cluster I, II-1/2/3) were absent in Cluster III (Fig. 4f, Cluster III). Notably, the weak correlation for actin in Cluster III found in the previous time lag correlation analysis (Fig. 4a) is consistent with the result of this pairwise time correlation analysis. This finding suggests that pairwise correlations at specific time points more precisely reveal the various aspects of the coordination between protrusion velocities and the underlying molecular dynamics.

Intriguingly, we did not identify a similarly significant instantaneous correlation between the protrusion velocity and Arp3 in any cluster (Fig. 4g). Conversely, we identified a significantly stronger instantaneous correlation between VASP intensities and protrusion velocities in all clusters in the time-specific correlation analysis (Fig. 4h). This is consistent with the previous study such that the edge velocity and lamellipodial VASP intensity were highly correlated when the leading edges of B16 melanoma cells had a uniform rate of protrusion^29^; however our study provided substantial quantitative evidence from the samples exhibiting highly heterogeneous and non-stationary edge movements. This further suggests that VASP compared to Arp2/3 plays a more direct role in controlling the protrusion velocity at the leading edge in all protrusion clusters. In Cluster I and II-1/2/3, VASP-dependent actin elongation tightly coordinates with Arp2/3 complex-mediated actin nucleation because actin exhibited a strong instantaneous correlation with protrusion velocity. Conversely, the significant and strong instantaneous correlation between VASP and the protrusion velocity in Cluster III begins to appear 100 s after protrusion onset (Fig. 4h, Cluster III), along with no correlation between actin and the protrusion velocity (Fig. 4f, Cluster III). This suggests that actin elongation by VASP plays a key role in the late phase of accelerating protrusion while Arp2/3 still plays a role in the early phase (Fig. 4i, Supplementary Note 2). We also demonstrated that VASP intensities contained sufficient information to predict protrusion phenotypes by the classification analysis (Fig. 4j, k, Supplementary Note 3).

Notably, both the strong correlation between VASP and the protrusion velocity observed in all clusters and the postulated mode of VASP in regulating accelerating protrusions suggest that VASP plays a more critical role in generating differential protrusion phenotypes. The differences in how VASP and Arp2/3 polymerize actin further validate our interpretation. VASP facilitates actin filament elongation by binding to the barbed ends of actin filaments at the leading edge^30–32^, whereas Arp2/3 binds to the sides of the mother filaments and initiates actin nucleation. Thus, the ability of Arp2/3 to directly control barbed end elongation is limited^33^. Because actin elongation at the barbed end pushes the plasma membrane and generates protrusion velocity, the strong correlation between VASP activity and protrusion velocity at the leading edge is plausible.

### Deconvolution of heterogeneous drug responses in protrusion

**Fig. 5:**
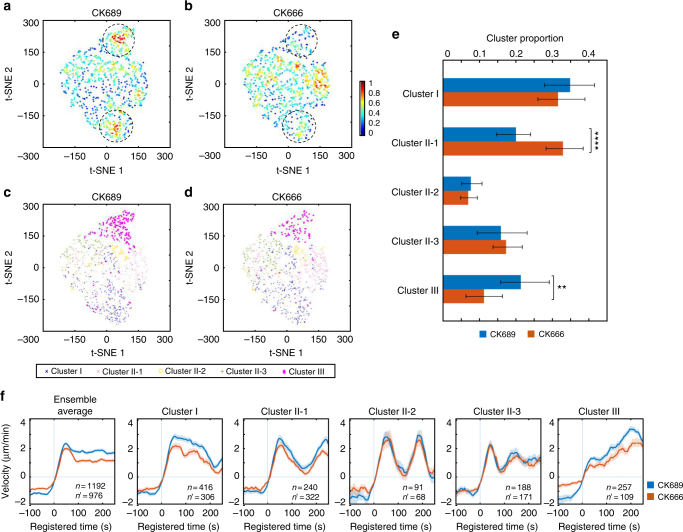
Functional validation by pharmacological perturbation of Arp2/3. **a**–**d** t-SNE plots of autocorrelation functions of protrusion velocity time series overlaid with the density of data (**a**, **b**) and cluster assignments (**c**, **d**). **e** Comparison of the proportion of each cluster between CK689 (50 μM, inactive control compound) and CK666 (50 μM). The error bars indicate 95% confidence interval of the mean of the cluster proportions. **p < 0.01 and ****p < 0.0001 indicate the statistical significance by bootstrap sampling. **f** Ensemble averaged velocity time series of entire samples and averaged velocity time series sampled in each cluster in CK689 or CK666-treated cells. All time series are registered with respect to protrusion onset (*t* = 0). Solid lines indicate population averages. Shaded error bands indicate 95% confidence intervals of the mean computed by bootstrap sampling. *n* and *n'* indicate the number of time series sampled in each cluster for CK689 and CK666, respectively. The numbers of cells used in the analysis are both 10 (CK689 and CK666)

Our statistical analyses thus far suggest that the early recruitment of Arp2/3 at the leading edge leads to VASP recruitment to barbed ends of actin filaments, giving rise to accelerating cell protrusion. Since Arp2/3 was implicated in the early phase of accelerating protrusion, we treated PtK1 cells with an Arp2/3-specific inhibitor, CK666^34^ (50 μM) to validate the functional role of Arp2/3. Notably, CK666-treated cells still exhibited highly active protrusion activities with 50 μM concentration, and they were visually indistinguishable from the control cells treated with the inactive compound, CK689. After pooling CK666 and CK689 data together, we performed the time series clustering analysis. CK666 and CK689-treated cells still exhibited similar temporal patterns in all clusters (Fig. 5f, Supplementary Fig. 6, Supplementary Movie 2 and 3), even if the protrusion velocities in Cluster I, II-1, and III were modestly reduced by CK666 (Fig. 5f, Cluster I/II-1/III). The t-SNE visualization of the ACFs of all protrusion time series revealed that CK666 (Fig. 5b) affected two densely populated areas in the control (CK689) cells (the dotted circles in Fig. 5a, b), and overlaying the cluster assignment in these t-SNE plots revealed that Cluster III was reduced by CK666 (Fig. 5c, d). The quantification of the proportion of each cluster confirmed that Cluster III was significantly reduced by the CK666 treatment (Fig. 5e, *p* = 0.0059, bootstrap sampling). In turn, this led to the significant increase of Cluster II-1 (Fig. 5e, *p* = 0.0001, bootstrap sampling). Intriguingly, the other clusters were not significantly affected by CK666, suggesting that the reduced Arp2/3 activities could be compensated by other actin regulators^35^. These results verify that Arp2/3 plays a specific functional role in accelerating protrusion. Furthermore, these demonstrate that our HACKS framework enables us to identify the susceptible clusters, which respond specifically to pharmacological perturbations.

**Fig. 6:**
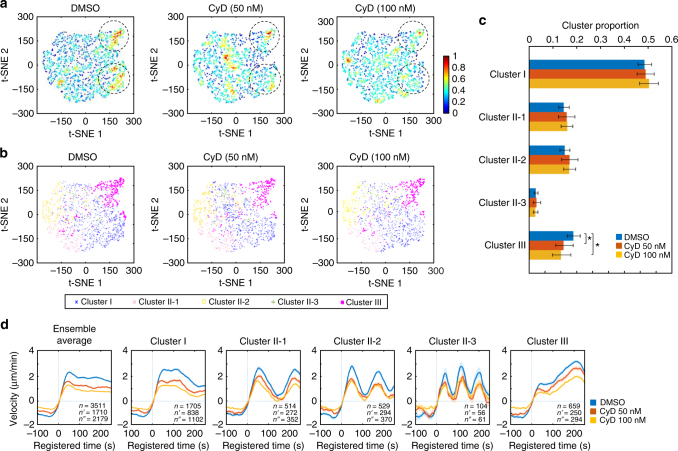
Functional validation by pharmacological perturbation of VASP. **a**,** b** t-SNE plots of autocorrelation functions of protrusion velocity time series overlaid with the density of data (**a**) and cluster assignments (**b**). **c** Dose-response of the proportions of clusters to CyD. The error bars indicate 95% confidence interval of the mean of the cluster proportions. *p < 0.05 indicates the statistical significance by bootstrap sampling. **d** Ensemble averaged velocity time series of entire samples and averaged velocity time series sampled in each cluster in DMSO or Cytochalasin D (50 or 100 nM)-treated cells. All time series are registered with respect to protrusion onset (*t* = 0). Solid lines indicate population averages. Shaded error bands indicate 95% confidence intervals of the mean computed by bootstrap sampling. *n, n'* and *n"* indicate the number of time series sampled in each cluster for DMSO, CyD 50nM, and CyD 100nM, respectively. The numbers of cells used in these analyses are 22 (DMSO), 16 (CyD 50 nM) and 20 (CyD 100 nM), respectively

Next, to validate the functional role of VASP in accelerating protrusion (Cluster III), we treated PtK1 cells with low concentrations (50 and 100 nM) of Cytochalasin D (CyD) to displace VASP from the barbed ends of actin filaments^36–39^. Using immunofluorescence, we confirmed that the CyD treatment effectively removed the phosphorylated VASP, which is a functional form of VASP, from the lamellipodial leading edge of PtK1 cells (Supplementary Fig. 7). Consistent with our previous correlation analyses where VASP intensities correlated with protrusion velocities in all clusters, the time series clustering analysis using the pooled DMSO and CyD (50, 100 nM) data revealed that protrusion velocities in all protrusion clusters in the CyD-treated cells were significantly reduced in a dose-dependent manner in comparison to DMSO-treated cells (Fig. 6d, Supplementary Fig. 8, Supplementary Movies 4–6). Nonetheless, the CyD-treated cells retained similar clustering structures, demonstrating the specificity of the CyD treatment in these low concentrations. The t-SNE plots of ACFs of each velocity time series also revealed that two dense areas were affected by the CyD treatment (the dotted circles in Fig. 6a), which includes the region of Cluster III (Fig. 6b). The proportion of Cluster III was significantly but modestly reduced by the CyD treatment (Fig. 6c, *p* = 0.043 for 50 nM, 0.018 for 100 nM, bootstrap sampling).

**Fig. 7:**
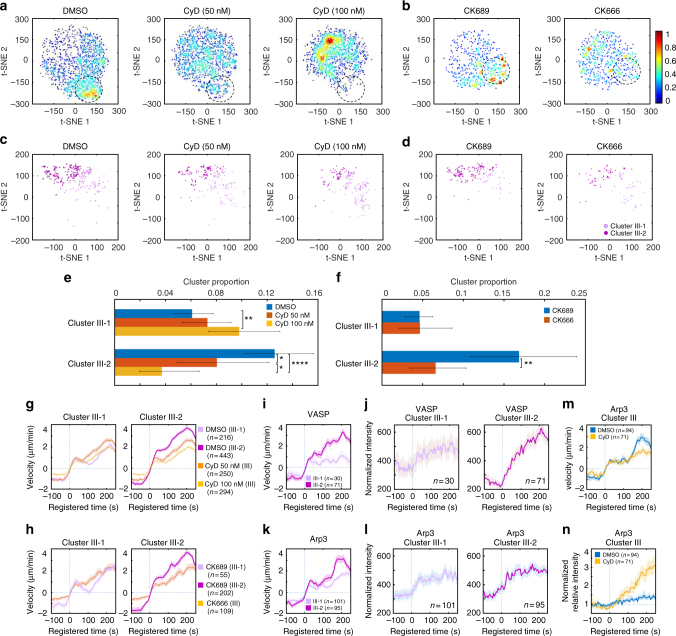
Functional validation of the differential coordination between Arp2/3 and VASP in strong accelerating protrusion. **a**, **b** t-SNE plots of the denoised protrusion velocity time series of the whole sample overlaid with the density of data. **c**,** d** t-SNE plots of the denoised velocities of the sub-clusters (Cluster III-1 and III-2) in Cluster III. **e**,** f** Comparison of the proportion of Cluster III-1 and III-2 upon Cytochalasin D treatment (**e**) or CK666 treatment (**f**). The error bars indicate 95% confidence interval of the mean of the cluster proportions. *p < 0.05, **p < 0.01, and ****p < 0.0001 indicate the statistical significance by bootstrap sampling. The numbers of cells used in this analysis are 22 (DMSO), 16 (CyD 50 nM), 20 (CyD 100 nM) and 10 (both CK689 and CK666), respectively. **g**,** h** Averaged velocity time series sampled in Cluster III (Cytochalasin D (**g**) or CK666 (**h**)), Cluster III-1 and Cluster III-2 (DMSO (**g**) or CK689 (**h**)). **i**–**l** Averaged velocity time series and normalized fluorescence intensity time series of Cluster III-1 and Cluster III-2 from unperturbed VASP-expressing cells (**i**, **j**) or Arp3-expressing cells (**k**, **l**). **m**, **n** Averaged velocity time series (**m**) and normalized fluorescence intensity time series of Cluster III (**n**) in Arp3-expressing cells upon treatment of DMSO or Cytochalasin D (100 nM). All time series are registered with respect to protrusion onset (*t* = 0). Solid lines indicate population averages. Shaded error bands indicate 95% confidence intervals of the mean computed by bootstrap sampling. *n* indicates the number of time series in each cluster

We observed CyD treatment tended to reduce the overall protrusion velocities. Therefore, we visualized the data distributions using t-SNE with denoised protrusion velocities instead of ACFs to further investigate the effects of CyD on Cluster III in terms of regulation of protrusion velocity. This t-SNE analysis revealed high-density regions of the subcellular protrusion velocities which are highly susceptible to the CyD and CK666 treatment (the dotted circles in Fig. 7a, b). Overlaying the cluster assignments in these t-SNE plots showed that Cluster III contained a substantial portion of the CyD and CK666-susceptible regions (Supplementary Fig. 9a). Notably, the t-SNE plots of Cluster III of the control cells (Supplementary Fig. 9b) suggest that Cluster III can be largely grouped into two, which may have differential susceptibilities to CyD and CK666. Therefore, we further divided Cluster III into two sub-clusters (Fig. 7c, d) based on denoised protrusion velocities pooled from CyD and CK666 data sets by a community detection algorithm^40^ (Supplementary Fig. 9c–e). While both Cluster III-1 and III-2 (Fig. 7g, h) maintained similar temporal patterns, Cluster III-2 had substantially stronger accelerating activities compared to Cluster III-1 (Fig. 7g, DMSO (Cluster III-1)/DMSO (Cluster III-2) and Fig. 7h, CK689 (Cluster III-1)/CK689 (Cluster III-2)). Intriguingly, the t-SNE plots revealed that "strongly accelerating protrusion" (Cluster III-2) was preferentially affected by the CyD (Fig. 7c) and CK666 (Fig. 7d) treatment. The quantification of the proportion of these sub-clusters (Fig. 7e, f) confirmed that strongly accelerating protrusion (Cluster III-2) was significantly reduced by the CyD treatment in comparison to DMSO treatment in a dose-dependent manner (*p* = 0.024 for 50 nM, <0.0001 for 100 nM, bootstrap sampling) (Fig. 7e, Cluster III-2), whereas the weakly accelerating protrusion (Cluster III-1) was increased (*p* = 0.006 for 100 nM, bootstrap sampling) (Fig. 7e, Cluster III-1). Therefore, the average protrusion velocities in Cluster III in CyD treatment were significantly reduced to be comparable to Cluster III-1 in DMSO treatment and was significantly lower than Cluster III-2 (Fig. 7g). Consistently, the proportion of strongly accelerating protrusion (Cluster III-2) was significantly reduced by the CK666 treatment (Fig. 7f, *p* = 0.0026, bootstrap sampling) and the average velocities of Cluster III in CK666 treatment were also reduced to those of weakly accelerating protrusion (Cluster III-1) in CK689 treatment (Fig. 7h). These data demonstrate HACKS allowed us to successfully identify the drug-susceptible sub-phenotypes, where strongly accelerating protrusion is specifically affected by inhibition of Arp2/3 or VASP.

Next, we further investigated whether dynamics of VASP and Arp3 in accelerating protrusion is differentially regulated between Cluster III-1 and Cluster III-2. We divided the intensity time series of VASP and Arp3 in Cluster III (Fig. 7j, l) into two sub-clusters and compared their differential dynamics. The recruitment dynamics of VASP in Cluster III-2 exhibited strong increase, while that of Cluster III-1 exhibited only moderate elevation, which is within the 95% confidence interval of the mean (Fig. 7j). On the other hand, Arp3 intensity patterns in Cluster III-1 and 2 were almost identical (Fig. 7l). This is consistent with our notion that Arp2/3 is involved in initiating accelerating protrusion and VASP is important in the output of accelerating protrusion. To functionally confirm this, we compared Arp3-GFP fluorescence dynamics at the leading edges in each cluster with or without 100 nM CyD treatment (Fig. 7m, n, Supplementary Fig. 10). To this end, we normalized Arp3 intensities at the leading edge by those of the lamella region in the same cell to quantitatively compare the Arp3 accumulation in different experimental condition. Under CyD treatment, the Arp3 fluorescence normalized by lamella intensity still started to increase at the protrusion onset in Cluster III (Fig. 7n). Normalized Arp3 fluorescence continued to increase up to four-fold more than the DMSO control while the protrusion velocity did not increase (Fig. 7m). First, this suggests that CyD treatment did not affect the initial Arp2/3 recruitment to the leading edge in accelerating protrusion, which proposes that Arp2/3 precedes VASP in accelerating protrusion. In addition, these data show that even increasing Arp2/3 recruitment under CyD treatment could not produce strongly accelerating protrusion without VASP activity. Therefore, the temporally ordered coordination between Arp2/3 and VASP is crucial to the strongly accelerating protrusion. Notably, such molecular temporal coordination was reported to be involved in cell protrusion^12,13,41,42^. Particularly, PI3K has been known to increase after protrusion onset to stabilize nascent cell protrusion^41^. Taken together, our HACKS framework combined with pharmacological perturbations effectively demonstrated that heterogeneous edge movements could be deconvolved into variable protrusion phenotypes to reveal the underlying differential regulation of actin molecular dynamics. We also successfully demonstrated that we could monitor the changes in actin regulator dynamics induced by functional perturbation.

## Discussion

We have demonstrated that our computational framework HACKS could effectively deconvolve heterogeneous subcellular protrusion activities into distinct protrusion phenotypes, establish an association between the each protrusion phenotype and the underlying differential actin regulator dynamics, and reveal specific phenotypes susceptible to pharmacological perturbations. Although previous studies have examined the spatiotemporal patterning of cell edge dynamics^11,43–45^, our study is the first to propose an effective framework to analyze the temporal heterogeneity in protrusion activities at the subcellular level and identify the subcellular protrusion phenotype. Together with the functional assays, we identified “strongly accelerating protrusion” susceptible to the pharmacological perturbations. Although previous studies also described persistent protrusion based on protrusion distance on longer time scales^11,13,37,46^, we first dissected protrusion phenotypes at fine spatiotemporal scales and quantitatively characterized persistently “accelerating protrusion”. Intriguingly, accelerating protrusion was later shown to be regulated by distinct actin regulator dynamics, although they accounted for a minor portion of the entire sampled protrusions. This finding indicates that identifying even a small subset of phenotypes is crucial to fully understand the mechanism underlying heterogeneous cellular behaviors.

We were also able to quantitatively measure how the underlying molecular dynamics are coordinated with protrusion phenotypes, thereby revealing the hidden variability of molecular regulatory mechanisms. Elucidating exact regulatory mechanisms related to protrusion heterogeneity has been difficult partly because it remains challenging to experimentally perturb a subset of molecules involved with specific subcellular phenotypes in situ. To address this challenge, our framework employed highly specific computational analyses. The result of our analyses provided quantitative and detailed information about the differential coordination between molecular dynamics and the protrusion phenotype at the subcellular level.

We also demonstrated that we could deconvolve the heterogeneity of drug responses of cellular protrusions using our HACKS framework by mapping protrusion time-series to two-dimensional phenotypic space using t-SNE and our time series clustering results. This approach revealed the protrusion phenotypes susceptible to pharmacological perturbations and functionally validated our hypothesis drawn from the statistical analysis: the temporally ordered coordination between Arp2/3 and VASP drives the accelerating protrusion. To date, the Arp2/3 complex has been widely accepted as a master organizer of branched actin networks in lamellipodia that acts by nucleating actin filaments^27^, whereas VASP has been thought to be an elongator of actin filaments or anti-capper of the barbed ends^30,31,35,47^. In this study, we focused on the distinct recruitment dynamics of Arp3 and VASP identified in the accelerating protrusion phenotype (Cluster III). This suggested that Arp2/3-dependent actin nucleation provides a branched structural foundation for protrusion activity, and VASP-mediated actin elongation subsequently takes over to persistently accelerate protrusions. Our functional studies using CK666 and Cytochalasin D confirmed that this coordination is critical to strongly accelerating cell protrusion and the recruitment timing and duration of Arp3 and VASP is finely regulated to generate differential protrusion activities. Notably, VASP was reported to increase cell protrusion activities^37,38,46^, and has been implicated in cancer invasion and migration^37,48,49^. Thus, the coordination of Arp2/3 and VASP may regulate the plasticity of protrusion phenotypes, and the functional deregulation of VASP or its isoforms in cancer may promote cellular migratory behaviors by promoting accelerating protrusion.

Furthermore, we consider HACKS is not limited to the analyses of subcellular protrusion heterogeneity: we anticipate that it can be expanded to study the morphodynamic heterogeneity of other types of cytoskeletal structures and membrane-bound organelles. Together with the further development of unsupervised learning along with an increased repertoire of molecular dynamics, we expect our machine learning framework to accelerate the mechanistic understanding of heterogeneous cellular and subcellular behaviors.

## Methods

### Local sampling and event registration

Using a custom-built software package^11,13^ written in MATLAB (MathWorks, MA, USA), we performed the following computational procedures. The threshold-based method was used to segment cell edges in the fluorescence images, and the cell edge velocity was calculated by tracking the cell edges using a mechanical model^11^. The software generated probing windows whose initial size was 500 nm by 500 nm along the cell boundary to locally sample the protrusion velocity and fluorescence intensity. The number of probing windows then maintained constant throughout the movie. The local protrusion velocity and fluorescence intensity were quantified by averaging the values within probing windows. By repeating this procedure in each frame of the time-lapse movies, we acquired the time series of protrusion velocities and fluorescence intensities.

We then identified significant protrusion events on a per-window basis. To reduce the effects of random fluctuations in the protrusion velocity time series, we obtained an edge displacement time series for a particular window by integrating the protrusion velocity over time. The noise of the time series was removed with a smoothing spline filter using the Matlab function csaps() with a smoothing parameter of 0.01. The small protrusion and retraction events considered insignificant in terms of the overall cell edge movement were further eliminated as follows. First, we identified local maxima/minima (protrusion/retraction onsets) at the edge displacement time series using the Matlab function findpeaks() and calculated the net protrusion/retraction distances for each event. A previous study using the same PtK1 cells showed that the distribution of distances could be decomposed into two exponential distributions, indicating small fluctuations and large movement during protrusion and retraction events^13^. Thus, small events whose protrusion distances were less than 720 nm (10 pixels in length) were discarded from the analysis. In addition, we eliminated short-term switches between the protrusion and retraction phases within 50 s. After these insignificant events were removed, the remaining protrusion onsets were used for event registration.

The protrusion velocity and fluorescence intensities over time in individual windows were registered by aligning the protrusion onset at *t* = 0. After the registration, the negative time indicates the retraction phase, and the positive time indicates the protrusion phase. Time series in negative time were limited by the preceding protrusion onset, and time series in positive time were limited by the subsequent retraction onset.

### Treating missing values

Because of image noise, the software could produce abnormal data in a rare case. In this case, the  values should be discarded from the time series. The following strategy was applied to treat these missing values: the entire time series was discarded if the number of continuous missing values was longer than eight. Otherwise, the average value of four values before and after the missing value was used to estimate the missing value.

### De-noising the samples by EMD

For each registered time series, the edge displacement was calculated from the edge velocity using the Matlab function trapz(). EMD^21^ was then applied to the transformed protrusion edge displacement to remove noise. Then, the denoised velocity was calculated from the denoised displacement using the Matlab function diff().

Cell edge movement is highly non-stationary. EMD^21^ is a local and data-driven de-noising method to decompose non-stationary signals into a series of intrinsic components. The general procedure of EMD can be described as follows:

1. Identify all minima and maxima of distance time series, *d*(*t*);
2. Connect the local maxima and minima respectively using an interpolation method to generate the envelope, e(*t*);
3. Compute the average of envelopes, ${\mathrm{avg}}\left( {t} \right) = [{e}_{{\mathrm{min}}}\left( {{t}} \right) + {e}_{{\mathrm{max}}}({t})]/2$;
4. Eliminate the average signal of the envelope from *d*(t) to obtain the residue: ${\mathrm{m}}\left(t\right) = {{d}}\left(t\right)-{\mathrm{avg}}\left( {{t}} \right)$;
5. Iterate from step (1) to (4) on the residue *m*(*t*) until the avg(*t*) becomes zero.

After EMD, the original signals can be decomposed into intrinsic mode functions without any loss of information, and the residue is called the trend. For each component, a de-trended fluctuation analysis is used to measure the self-affinity as the fractal scaling index (*α*), which estimates the fractal-like autocorrelation properties. The value of *α* is inversely related with the possibility that the component is originated from noise. In our procedure, the code was obtained from the previous publication^50^, and the value of α was empirically set to 0.33.

### Determining the time interval for the clustering analysis

The duration of cell protrusion is heterogeneous, and some protrusion events are not completely recorded because of the finite length of the movies. Our clustering analysis focused on the time series with equal temporal length. Therefore, the time series shorter than a certain temporal length were discarded from the analysis. We estimated the optimal temporal length by maximizing the multiplication of the number of samples and the temporal length. By optimizing these two factors, the best temporal length was approximately 50 and more than 60% of the time series was retained for the further analysis. Moreover, five frames before the protrusion onset were also included for the further analysis. Therefore, the time series for the analysis consisted of 56 frames including the previous five frames before the protrusion onset and 51 frames after the protrusion onset.

### Representing the velocity by SAX

In order to extract the relevant features related to shape patterns of time series in high dimensions, we applied SAX^51^ to our time series dataset to reduce dimensionality and discretize the data. The general procedure of SAX is summarized as follows:

1. Manually determine the reduced dimension, *N*, and the symbolic number, *M* (the number of discretization levels).
2. The time series data across the entire time range are pooled together and fitted to a Gaussian distribution. Then the entire time series were discretized into *M* levels with equal probabilities using the fitted Gaussian distribution. Each level was represented by a pre-defined symbol.
3. The time series was divided into *N* intervals along the time. The average value was calculated in each interval to represent the raw time series.
4. The pre-defined symbols obtained in (2) were assigned to each interval based on the averge value calculated in (3).
5. Iterate from step (2) to (4) until all samples are represented.

After the SAX representation, all time series data were reduced to low-dimensional (*N*) symbolic series data. In this analysis, *M* was set to four and *N* was set to 16. Here, four symbols that range from zero to three were used to calculate the autocorrelation coefficients. In addition, the symbolic representation process in SAX also removed noise due to local averaging effects.

### Calculating the sample dissimilarity

To measure the dissimilarity of two time series, the original description of SAX representation proposed an approximate Euclidean distance of SAX as a dissimilarity measure^22^. Instead, we used the dissimilarity measure based on the estimated ACFs^52^. First, the estimated autocorrelation vector was calculated, and the squared Euclidean distance between the autocorrelation coefficients was then used to measure the dissimilarity of two velocity time series X and Y as follows:

$$d^2_{{\mathrm{ACF}}}(X,Y) = \mathop {\sum }\limits_{i = 1}^L (ACF(X)_i - ACF(Y)_i)^2.$$

In our implementation, the ACF distance was calculated using the TSdist R package^53^. In order to evaluate the requirement of the ACF distance in our clustering analysis, we compared the clustering performance using different dissimilarity measures in Supplementary Fig. 1.

### Clustering the velocities by Density Peak

After we calculated the pairwise dissimilarity of the time series, we performed a clustering analysis using the Density Peak clustering algorithm^23^. It is desirable that cluster centers have local density maxima and are separated from other dense regions in feature space. Based on this notion, Density Peak can generate a density–distance map that can be used to determine the optimal number of clusters and cluster centers. In addition, Density Peak can build up hierarchical tree structures of clusters by linking the samples with higher density but lower distance. Based on the selected number of clusters and cluster centers, the samples in the hierarchical tree can be divided into several clusters.

The procedure to generate the density–distance map was as follows: Each sample was represented by two parameters: local density and minimum distance. The local density of each sample was estimated by the crowdedness of samples in its neighboring region. The minimum distance was the distance of the closest samples with higher density. By plotting these two parameters in two dimensions, we built up the density–distance map. Based on the definition, the samples with a high density and distant from other samples with higher density were localized in the upper-right region of the density–distance map. Therefore, the sparse samples in the upper-right region were selected as cluster centers visually, and the number of these cluster centers were determined as the number of clusters. Finally, the hierarchical tree was divided into several disconnected sub-trees as clusters.

In our implementation, the density around each sample was determined by calculating the sum of distances with the Gaussian Kernel of the manually selected radius as follows:

$$\rho \left( {S_i} \right) = \mathop {\sum }\limits_{k = 1,i \ne k}^N e^{( - \frac{{d\left( {S_i,S_k} \right)}}{{\mathrm {dc}}})^2}$$

Here, dc was selected by a grid search method of the range of the sample dissimilarity to get the good performance of density–distance map. The number of clusters was manually selected by the visual inspection of the density–distance map. Moreover, in order to further confirm the number of clusters suggested by the density–distance map, we also applied three criteria: DBI, Average Silhouette and Calinski–Harabasz pseudo *F*-statistic to evaluate the number of clusters implemented in ClusterSim Package^54^.

### Validating clustering results

We used the following methods to validate our clustering results.

Ordered dissimilarity map: The distances between samples within the same clusters should be smaller than those between samples in different clusters. Therefore, after the samples were grouped by cluster indices and ordered by dissimilarity, the distance map can be visualized as blocks along the diagonal. In addition, this method is particularly suitable for Density Peak clustering because the required input of the density peak clustering method is a dissimilarity matrix.

MDS: Classical MDS^55^ is a method to visualize the similarity of individual samples in a data set based on the distance dissimilarity matrix. The MDS algorithm aims to place each sample in a lower dimensional space under the constraint that the between-sample distances are preserved as much as possible. Here, we used the Matlab function, cmdscale().

t-SNE: t-SNE (t-distribution Stochastic Neighboring Embedding)^56^ is an advanced dimensionality reduction technique and particularly suitable for the visualization of high-dimensional datasets. In t-SNE, the probability distribution of paried samples in original high-dimensional spaces is constructed to represent the similarity between the samples. Then, t-SNE attempts to find the similar distribution of paired samples in the low-dimensional space by minimizing the Kullback-Leibler (K-L) divergence between these two distributions. Here, the parameters (final dimension, initial-dimension, perplexity) of t-SNE were (2, 10, 20), meaning that the data set was first reduced to ten dimensions and then mapped to two dimensions by optimizing the K–L divergences.

Silhouette plot: Silhouette plots^57^ were used to validate the consistency within clustered data. For each sample *i*, *a(i)* represents the average dissimilarity within the same cluster, whereas *b(i)* represents the lowest dissimilarity with the sample from any other clusters. The silhouette value of the sample *i*, is calculated as follows:

$$s\left( i \right) = \frac{{b\left( i \right) - a(i)}}{{\max (b\left( i \right),a\left( i \right))}}.$$

The range of *s(i)* is [−1,1], and larger values indicate better clustering performance.

### Normalizing Actin, Arp2/3, and VASP fluorescence signals

Because differences in the expression levels of fluorescent proteins and their endogenous non-fluorescent proteins are not known, we cannot average the registered time series of raw fluorescence intensity. Moreover, we aimed to determine the recruitment pattern for a fluorescent protein independent of the absolute level. Therefore, before a protrusion event was registered, we separately normalized the intensity time series of each window by min-max scaling as follows:

$$I_{\rm norm}\left( {w,t} \right) = \frac{{I\left( {w,t} \right) - \min \left( {I\left( {w,t} \right)} \right)}}{{\max (I\left( {w,t} \right)) - \min ({\mathrm{I}}\left( {w,t} \right))}} \ast 1000.$$

### Correlation analysis

The time lag correlation analysis between two time series as a function of time lag is used to discover the temporal relationship^12^. Pearson’s correlation coefficients were calculated using the time series of only protrusion segments (after protrusion onsets). The 95% confidence intervals for the average correlation were calculated by bootstrap resampling (Matlab function bootci()).

The time-specific correlation analysis between two activities (velocity and regulator intensity) is used to exploit their temporal variation^13^. After the protrusion velocity and fluorescence intensities were registered with respect to protrusion onset at *t* *=* *0*, Pearson’s correlation coefficients (Matlab function corrcoef()) between the fluorescence intensity at *t*_1_ and protrusion velocity at *t*_2_ across the samples were calculated across the time points, where *t*_1_ and *t*_2_ were measured relative to the protrusion onset. Two sample K–S (Kolmogorov–Smirnov) test was used to test the significance of the maximum correlation value in time lag correlation and Benjamini–Hochberg procedure for controlling the false discovery rate (FDR) was used to show the significance of the time-specific correlation.

### Clustering analysis of drug-treatment data

When we compared the proportions of each cluster with and without drug treatment, we pooled the registered protrusion velocity time series from the control and drug-treated experiments to maintain the same cluster boundaries, and then applied our time series clustering to them under the same clustering criteria. For DMSO/CyD treatment, the parameter of Density Peak cluster was 0.71. For CK689/CK666 treatment, the parameter of Density Peak cluster was 0.46.

For the GFP-Arp2/3 experiments, where we compared the temporal patterns in the similar clusters, we applied our time series clustering to the control and CyD treatment experiment individually. The parameters of Density Peak cluster were both 0.71.

### Identification of the drug-sensitive phenotypes

We pooled the control and drug-treatment data and visualized the data distribution of denoised velocity time series using t-SNE^56^. The initial dimension and the perplexity of the t-SNE were 30 and 50. Using the t-SNE plots, we visually identified the drug-susceptible regions where the data from the drug treatment were depleted in comparison to the control. By overlaying the cluster assignments, we identified which clusters were mainly affected by the drug treatment. We extracted the data belonging to these drug-susceptible clusters and applied community detection method^40^ to identify the sub-clusters. Then we merged these sub-clusters into two clusters based on the magnitude of the average velocity. In addition, the boundaries of the drug-susceptible regions were considered to finalize the sub-cluster structure. Finally, the cluster proportions with and without drug treatment were compared to validate the drug-sensitive phenotype.

### Statistical testing of the proportions of drug-sensitive phenotypes

We quantified the drug effect based on the cluster proportion. We counted the number of each cluster in each cell for the control and drug treatment experiments. These numbers in each cell were resampled using bootstrp() in MATLAB to build 10,000 different bootstrapped data set, and the distribution of the proportion of each cluster in each experimental set was created. Using these distributions, *p*-values were calculated by estimating the probability that the cluster proportion of one experiment was greater or less than that of the other experiment (one-tailed test). The 95% confidence intervals of the proportions were estimated by the Matlab bootci() function.

### Spatial distribution of subcellular protrusion clusters

We calculated the conditional probability that the samples within the same cluster co-existed over the distance as follows. In each iteration, we randomly selected eight movies from the total 36 movies and then sampled 40 frames in each movie. For a certain distance or window gaps, *k*, we calculated the five-by-five occurrence matrices, $M_k(cl_i,cl_j)$ for different pairs, $cl_i,cl_j$ of five clusters without considering the direction along cell edges. Based on the occurrence matrix, we calculated the conditional probability of each cluster pair for different window gaps as follows.

$$p_k\left( {cl_i|cl_j} \right) = \frac{{M_k(cl_i,cl_j)}}{{\mathop {\sum}\nolimits_{cl_i} {M_k\left( {cl_i,cl_j} \right)} }},\,cl_i,cl_j = 1, \ldots ,5.$$

We averaged the conditional probability $p_k\left( {cl_i|cl_j} \right)$with 500 iterations and the 95% confidence intervals of the mean was estimated by bootstrapping (bootci() in Matlab).

### Evaluating different time series clustering methods

To show the effectiveness of our time series clustering, three main components, SAX for dimensional reduction, ACF for dissimilarity measure and Density Peak for clustering, were evaluated by replacing them with different methods as follows.

1. Evaluating the role of SAX: Without SAX, ACF was directly applied to the denoised velocity data set to calculate the ACF distances, implemented in the TSdist R package^53^. The Density Peak method was then used for clustering with the cut-off distance parameter, 0.61. Community detection was used for clustering with the number of neighbors, 80.
2. Evaluating the role of ACF distance: The dissimilarity measure was changed from the ACF distance to the distance metric proposed by SAX, which was the lower bound of the true Euclidean distance^51^. Here, eight was empirically selected as the number of symbols for SAX, and eight symbols ranging from 0 to 7 were used to calculate the dissimilarity. The cut-off distance of the Density Peak clustering was 0.46.
3. Evaluating the role of the combination of SAX and ACF: Without dimensional reduction by SAX, the denoised velocity data set was directly used to calculate the dissimilarity using the Euclidean distance from the TSdist R package. The Density Peak method was then used for clustering, and the cut-off distance for the Density Peak method was 0.46.
4. Evaluating the role of Density Peak clustering: Instead of Density Peak clustering, a conventional clustering method, *k*-means, and community detection were used for comparison while all other steps remained unchanged. Since the number of clusters for *k*-means should be determined first, two criteria DBI^24^ and Silhouette criteria^57^ were used to identify the number of clusters. In our analysis, the number of clusters for *k*-means was set to the optimal number *K* = 7. Community detection was also applied for comparison using the number of neighbors, 300 or 350 to generate six or five clusters respectively.

### Classification analyses of actin regulator intensities

To further investigate the role of VASP in accelerating cell protrusions, we applied the classification approach to the fluorescence intensity time series with their corresponding protrusion clusters. For this purpose, we focused on the classification between the non-accelerating protrusion class (Clusters I/II) and accelerating protrusion class (Cluster III). First, the fluorescence intensity time series was normalized to have a mean of zero and a standard deviation of one for each window. We used three different classification algorithms to measure the performance of the classification, including random forest (RF)^58^, support vector machine (SVM)^59^, and deep neural networks (DNN)^60^. The inputs of the classifiers were the normalized fluorescence intensities of selected frame intervals based on protrusion onset previously, and the output was the corresponding protrusion class (non-accelerating vs accelerating protrusion). The supervised learning was performed using the Python Scikit-Learn toolkit for RF and SVM^61^ and Keras with Theano engines in Python for DNN. Because the number of time series in the non-accelerating protrusion class (Clusters I/II) was larger than those of the time series in the accelerating protrusion class (Cluster III), we under-sampled the accelerating protrusion class so that the number of data points in two classes had the same. For reproducible results, random under-sampling was applied ten times to the non-accelerating protrusion class using the Imbalanced-learn package^62^. Cross-validation was performed with 67 and 33% splitting of each sample data set for training and testing. Moreover, the cross-validations were repeated ten times after randomly shuffling the data in each iteration. Hence, we performed the training procedures for each fluorescence intensity data set for 100 times. To assess the performance of the classification, we used accuracy (*N*_c_*/N*), where *N* is the number of total time series and *N*_c_ is the number of the correctly predicted time series, and Matthews correlation coefficient (MCC) defined as:

$$\frac{{N_{\mathrm{tp}}N_{\mathrm {tn}} - N_{\mathrm {fp}}N_{\mathrm {fn}}}}{{\sqrt {(N_{\mathrm{tp}} + N_{\mathrm {fp}})(N_{\mathrm{tp}} + N_{\mathrm {fn}})(N_{\mathrm {tn}} + N_{\mathrm {fp}})(N_{\mathrm {tn}} + N_{\mathrm {fn}})} }}.$$

Here, *N*_tp_*, N*_tn_*, N*_fp_, and *N*_fn_ are the numbers of true positives, true negatives, false positives and false negatives, respectively. The accuracy and MCC were calculated using the Python Scikit-Learn toolkit, where the parameters used in three classifiers are shown in Supplementary Table 5, which were determined by a grid search approach.

### Normalizing GFP-Arp2/3 fluorescence signals

In order to quantitatively compare the fluorescence intensity of GFP-Arp2/3 between DMSO and CyD-treated cells, we normalized the Arp3 intensity time series in each cell as follows to make sure that the normalized intensities of protrusion onset in these two cases were similar. In each cell, we manually selected the lamella regions, which did not contain bright fluorescence spots. Then, we calculated the average fluorescent intensity, *I*_la_ in these regions. We also selected background region outside the cell and the average background intensity, *I*_b_ was calculated. Finally, we calculated the GFP-Arp2/3 fluorescent normalized intensity value, *I*_norm_ from the raw intensity, *I* in each cell as $I_{\mathrm {norm}} = (I - I_{\mathrm {b}})/(I_{\mathrm {la}} - I_{\mathrm {b}})$.

### General statistical methods

The sample size was determined as follows. We generally used more than 100 probing windows from multiple cells (see individual figures or figure legends). The number of the probing windows was determined to be sufficient when the averaged time series displayed a distinct pattern with variations that substantially exceeded the 95% confidence interval.

Inclusion/exclusion of samples was determined as follows. We visually examined cellular morphology, the level of protein expression, and the number of nuclei in each cell movie. We performed our analysis using the cells with a flat, minimally ruffling morphology and wide leading edges, low expression level of fluorescent proteins, and single nucleus. At this stage, we did not know the cluster distribution along the cell edges and how the protein dynamics would behave. Thus, this data selection can be assumed unbiased for the presented analyses.

Justification of statistical tests: we used two-sample K-S (Kolmogorov–Smirnov) test implemented with the Matlab function kstest2() for statistical testing. The K-S test does not assume the distribution of the data. The variances of the data between groups were similar (See each figure). For multiple hypothesis testing in time-specific correlation analysis, Benjamini-Hochberg procedure for controlling the FDR was used to provide stronger control of the family-wise error rate. The 95% confidence interval of the velocity and normalized intensity was calculated using the bootstrap Matlab function bootci(), and the number of bootstrap samples was set to 1000.

### Cell culture and drug treatment

Cell culture and live cell imaging procedures were followed according to the previous studies^13^. All imaging was performed in imaging medium (Leibovitz’s L-15 without phenol red, Invitrogen) supplemented with 10% fetal bovine serum (FBS), 0.1 mg ml^−1^ streptomycin, 100 U ml^−1^ penicillin, 0.45% glucose, 1.0 U ml^−1^ Oxyrase (Oxyrase Inc.) and 10 mM Lactate. Cells were then imaged at 5 s intervals for 1000 s using a 60 × , 1.4 NA Plan Apochromat objective for spinning disk confocal microscopy.

PtK1 cells were cultured in Ham’s F12 medium (Invitrogen) supplemented with 10% FBS, 0.1 mg ml^−1^ streptomycin, and 100 U ml^−1^ penicillin. For the characterization of actin regulator dynamics (Figs. 2 and 3), cells were transfected with one of the DNA constructs including HaloTag-VASP (N-term), HaloTag-Arp3 (C-term), SNAP-tag-Actin, and empty HaloTag by electroporation using Neon transfection system (Invitrogen) according to the manufacturer’s instructions (1 pulse, 1400 V, 20 ms) and were grown on acid-washed glass #1.5 coverslips for 2 days before imaging. Prior to imaging, expressed HaloTag or SNAP-tag fusion proteins were labeled with HaloTag-TMR ligand (Promega) or SNAP-tag-TMR (New England BioLabs) ligand according to the manufacturer’s instructions. PtK1 cells were acquired from Gaudenz Danuser lab. They were routinely tested for mycoplasma contamination.

MCF10A cells were cultured in low‐glucose DMEM:Ham’s F12 nutrient media supplemented with 5% horse serum, 10 mM HEPES pH 7.4, and a growth factor cocktail including 20 ng ml^−1^ EGF, 10 μg ml^−1^ insulin, 0.5 μg ml^−1^ hydrocortisone, and 100 ng ml^−1^ cholera toxin. Cells were grown on 27-mm glass bottom dishes (Thermo Scientific, cat. #150682) for 2 days. Cells were serum starved for 24 h and stimulated with growth media containing 10% horse serum before imaging. For plasma membrane staining, cells were incubated with 5 μg ml^−1^ CellMask Orange (Invitrogen) for 5 min followed by manufacturer’s protocol. MCF10A cells were acquired from Joan Brugge lab. They were routinely tested for mycoplasma contamination.

For the drug treatment experiments (Figs. 5–7), PtK1 cells were grown on 27-mm glass bottom dishes (Thermo Scientific, cat. #150682.) for 2 days and stained with 5 μg ml^−1^ CellMask Deep Red (Invitrogen) following manufacturer’s protocol. GFP-Arp3 expressing PtK1 cells were further selected by G418 before imaging. For Arp2/3 inhibition experiments, cells were incubated with 50 μM of CK666 or CK689 (EMD Millipore) for an hour before imaging. For Cytochalasin D experiments, cells were incubated with DMSO or Cytochalasin D (Sigma) for half an hour before imaging.

### Light microscopy

All microscopy was performed using the set up as follows: Nikon Ti-E inverted motorized microscope (including motorized focus, objective nosepiece, fluorescence filter turret, and condenser turret) with integrated Perfect Focus System, Nikon Plan Apo 1.4 NA DIC optics (60×), Yokogawa CSU-X1 spinning disk confocal head with manual emission filter wheel with Spectral Applied Research Borealis modification, Spectral Applied Research custom laser merge module (LMM-7) with AOTF and solid state 445 nm (200 mW), 488 nm (200 mW), 514 nm (150 mW), 561 nm (200 mW), and 637 nm (140 mW) lasers, Semrock 405/488/561/647 and 442/514/647 dichroic mirrors, Ludl encoded XY stage, Ludl piezo Z sample holder for high speed optical sectioning, Prior fast transmitted and epi-fluorescence light path shutters, Hamamatsu Flash 4.0 LT sCMOS camera, 37 °C microscope incubator enclosure with 5% CO_2_ delivery (In Vivo), Molecular Devices MetaMorph v7.7, TMC vibration-isolation table.

### Immunofluorescence

PtK1 cells were seeded on cover slips coated with poly-D-lysine. Prior to fixation, cells were incubated with 100 nM DMSO or Cytochalasin D for 30 min. After drug treatment, cells were fixed with 4% paraformaldehyde in PBS, permeabilized by incubation with 0.1% Triton X-100 in PBS, and subsequently blocked with 1% BSA in PBS for 1 h. To verify the cellular localization of p-VASP and F-actin, cells were incubated with mouse anti-p-VASP antibody (Santa Cruz, sc-365564) and Alexa Fluor 647 Phalloidin (ThermoFisher, A22287) 1 h in the dark. The cells were washed with PBS for three times and incubated with anti-mouse Alexa Fluor 488 (Invitrogen) for 1 h in the dark. The Ptk1 cells were subsequently washed with PBS for three times and mounted with Gold antifade reagent (Invitrogen). Imaging was performed using the same spinning disk confocal microscope.

### Plasmid construction

Mouse VASP was subcloned into pFN21A vector (Promega) containing an N-terminal fusion to HaloTag. Human Arp3 was subcloned into the pFC14K vector (Promega) containing a C-terminal fusion to HaloTag according to the manufacturer’s instructions. A SNAP-tag-actin in C1-vector with a truncated CMV promoter (kindly provided by Martin Schwartz) was used. GFP-Arp3 was a gift from Matthew Welch (Addgene plasmid # 8462).

### Code availability

The code used in the current study is available from the corresponding author upon reasonable request.

### Data availability

The data sets used in the current study are available from the corresponding author on reasonable request.

## Electronic supplementary material

Supplementary Information

Description of Additional Supplementary Files

Supplementary Movie 1

Supplementary Movie 2

Supplementary Movie 3

Supplementary Movie 4

Supplementary Movie 5

Supplementary Movie 6
