# Supplementary material for: Deconvolution of subcellular protrusion heterogeneity and the underlying actin regulator dynamics from live cell imaging
Source: Nat Commun. 2018 Apr 27;9:1688. doi: 10.1038/s41467-018-04030-0 (PMC5923236; doi:10.1038/s41467-018-04030-0)
Supplement: Supplementary file 1 — Supplementary Information [file 41467_2018_4030_MOESM1_ESM.pdf]

Supplementary Information

Deconvolution of subcellular protrusion heterogeneity and the underlying actin regulator dynamics from live cell imaging

Wang et al.

	Global		Cluster I		Cluster II-1		Cluster I-2		Cluster II-3		Cluster III	
	#Video	#Window	#Video	#Window	#Video	#Window	#Video	#Window	#Video	#Window	#Video	#Window
All (=Velocity)	36	2756	34	764	35	367	35	625	36	674	33	326
Actin	10	934	10	215	10	134	10	255	10	245	10	85
Arp3	11	757	11	204	11	112	11	161	11	178	10	102
VASP	9	682	8	242	9	85	8	117	9	137	7	101
HaloTag	6	383	5	103	5	36	6	92	6	114	6	38

Supplementary Table 1. Number of cells and probing windows used in the time series clustering analysis

Method combination				Persistent pattern	Three oscillating patterns
DV	SAX	Distance	Clustering method		
Yes	No	ED	DP	No	No
Yes	No	ED	CD	No	No
Yes	No	ACF	DP	Yes	No
Yes	No	ACF	CD	Yes	Yes
Yes	Yes	app_ED	DP	No	No
Yes	Yes	ACF	*k*-means	Yes	Yes
Yes	Yes	ACF	CD	Yes	Yes
Yes	Yes	ACF	DP	Yes	Yes

DV: De-noised velocity
ED: Squared Euclidean distance
app_ED: Approximate Euclidean distance in SAX
ACF: Squared Euclidean distance based on autocorrelation
SAX: Symbolic aggregate approximation
k-means: *k*-nearest mean method
DP: Density peaks
CD: Community detection

Supplementary Table 2. Summary of the results from the differential combinations of algorithms in time series clustering

	Cluster I	Cluster II-1	Cluster II-2	Cluster II-3	Cluster III
Actin-Arp3	3.47×10^{-3}	0.041	2.36×10^{-4}	3.64×10^{-8}	0.075
Arp3-VASP	8.77×10^{-17}	1.55×10^{-10}	3.90×10^{-9}	5.25×10^{-9}	3.87×10^{-14}
Actin-VASP	7.62×10^{-13}	1.16×10^{-5}	2.15×10^{-10}	2.86×10^{-3}	5.16×10^{-12}

Supplementary Table 3. The *p*-values for statistical analyses of the maximum correlation coefficients from time-lag correlation analysis presented in Figure 4e All *p*-values were calculated by two-sample two-tailed Kolmogorov-Smirnov (KS) test.

	SVM			DNN			RF		
Protein pair	Actin-Arp3	Actin-VASP	Arp3-VASP	Actin-Arp3	Actin-VASP	Arp3-VASP	Actin-Arp3	Actin-VASP	Arp3-VASP
Accuracy	2.21×10^{-08}	5.06×10^{-30}	1.32×10^{-38}	0.047	1.27×10^{-28}	8.66×10^{-34}	0.003	2.96×10^{-32}	5.77×10^{-37}
MCC	2.21×10^{-08}	1.16×10^{-26}	3.70×10^{-36}	0.14	1.27×10^{-28}	9.30×10^{-31}	0.003	5.11×10^{-33}	2.33×10^{-35}

SVM: Support vector machine
DNN: Deep neural network
RF: Random forest
MCC: Matthews correlation coefficient

Supplementary Table 4. Statistical analyses of accuracy and Matthews correlation coefficients from the classification analysis presented in Figure 4j-k The *p*-values of the differences in accuracy and Matthews correlation coefficients between the protein pairs from the classification analysis of Cluster III against Clusters I and II-1/2/3. All *p*-values were calculated by two-sample two-tailed Kolmogorov-Smirnov (KS) test.

Methods	Hyperparameters	VASP	Arp2/3	Actin
RF	N_estimators	100		
	OOB_score	True		
SVM	C	1.93		
	γ	0.037	0.014	0.037
DNN	1st layer	Convolution, 3 filters with 3 taps, ReLU activation		
	2nd layer	Dense, 30 taps, 20% dropout, ReLU activation		
	3rd layer	Dense, 10 taps, 20% dropout, ReLU activation		

SVM: Support vector machine
DNN: Deep neural network
RF: Random forest

Supplementary Table 5. Hyperparameters used in the classification methods for each protein case. In random forest (RF), *N_estimaters* indicates the number of trees in the forest. *oob_score (true)* means to use out-of-bag samples to estimate the generalization accuracy. In support vector machine (SVM), the parameter C shows the penalty of the error term while γ represents the kernel coefficient. In deep neural network (DNN), there are three layers. Other non-specified parameters are set to the default parameters in Scikit-Learn and Keras package.

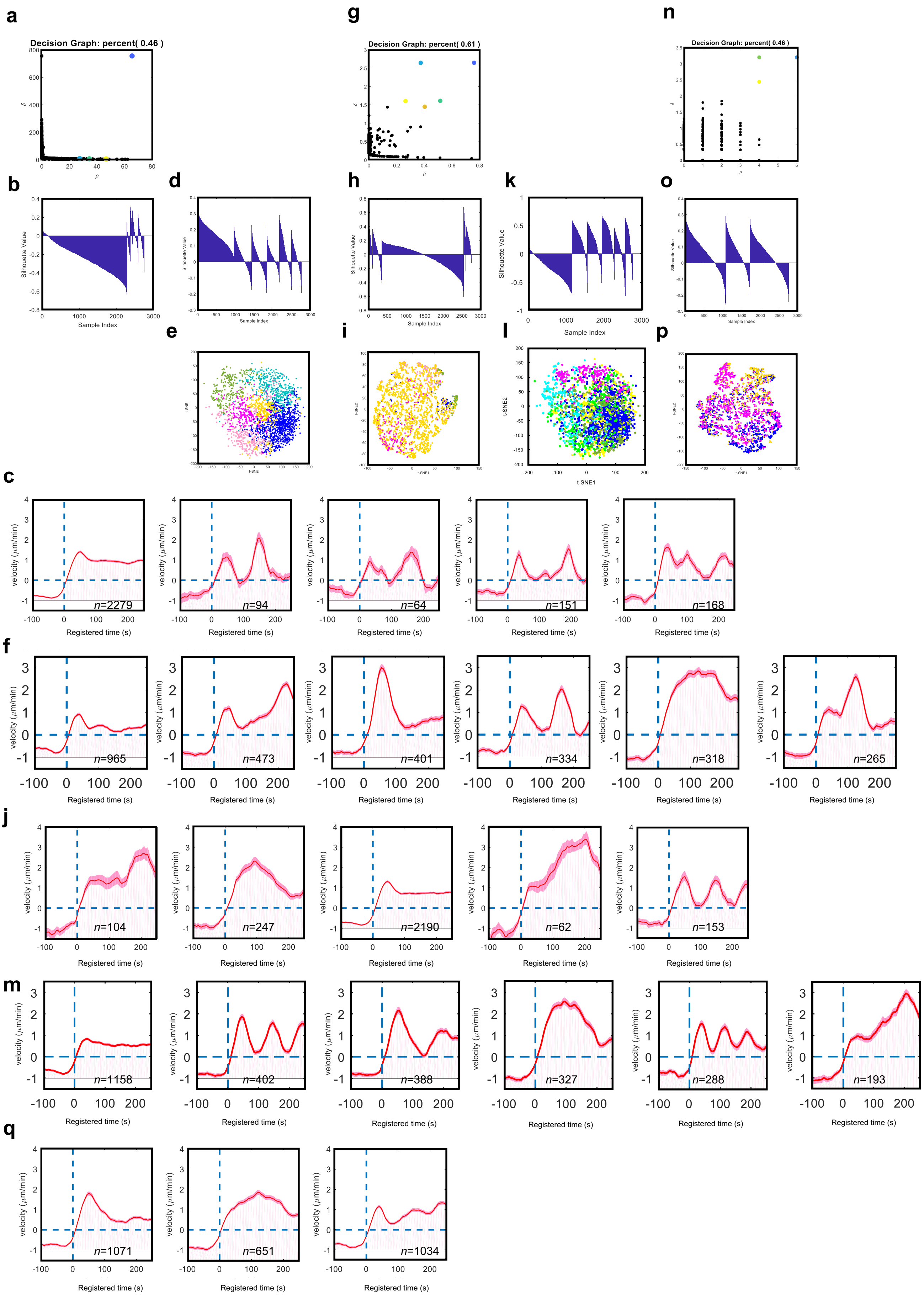

Supplementary Figure 1. Evaluating the role of ACF distance (continued)

Supplementary Figure 1. (continued) (a-c) The density peak clustering results using Euclidean distance without SAX. **(d-f)** The community detection results using Euclidean distance without SAX. **(g-j)** The density peak clustering results using ACF distance without SAX. **(k-m)** The community detection results using ACF distance without SAX. (**n-q)** The density peak clustering results using app_ED distance with SAX. (a, g, n) density map, (b, d, h, k, o) silhouette plot, (e, i, l, p) t-SNE plot. (c, f, j, m, q) protrusion velocity profiles in each cluster. (*n:* the number of protrusion segments used for the analysis, images from 36 cells were used for the analysis.)

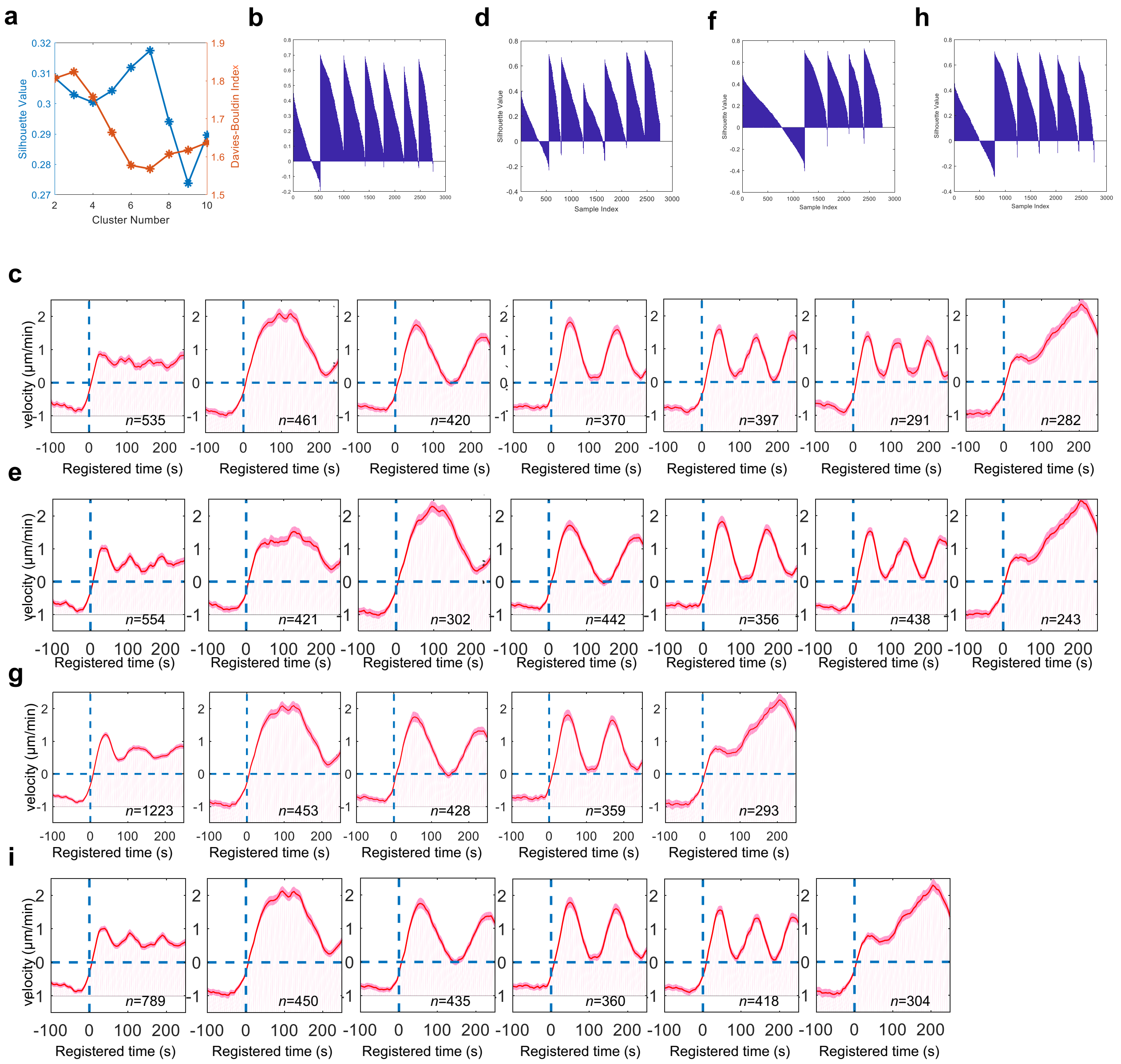

Supplementary Figure 2. Evaluating the roles of SAX and density peak clustering (a-e) The *k*-means clustering results using ACF distance with SAX. (a) Estimating the optimal number of clusters using DBI and silhouette value. (d-e) The result from another trials of *k*-mean. **(f-i)** The community detection results using ACF with SAX, (f-g) with five cluster condition and (h-i) with six cluster condition. (b, d, f, h) silhouette plots and (c, e g, i) protrusion velocity profiles of clusters. (*n*: the number of protrusion segments used for the analysis, images from 36 cells were used for the analysis.)

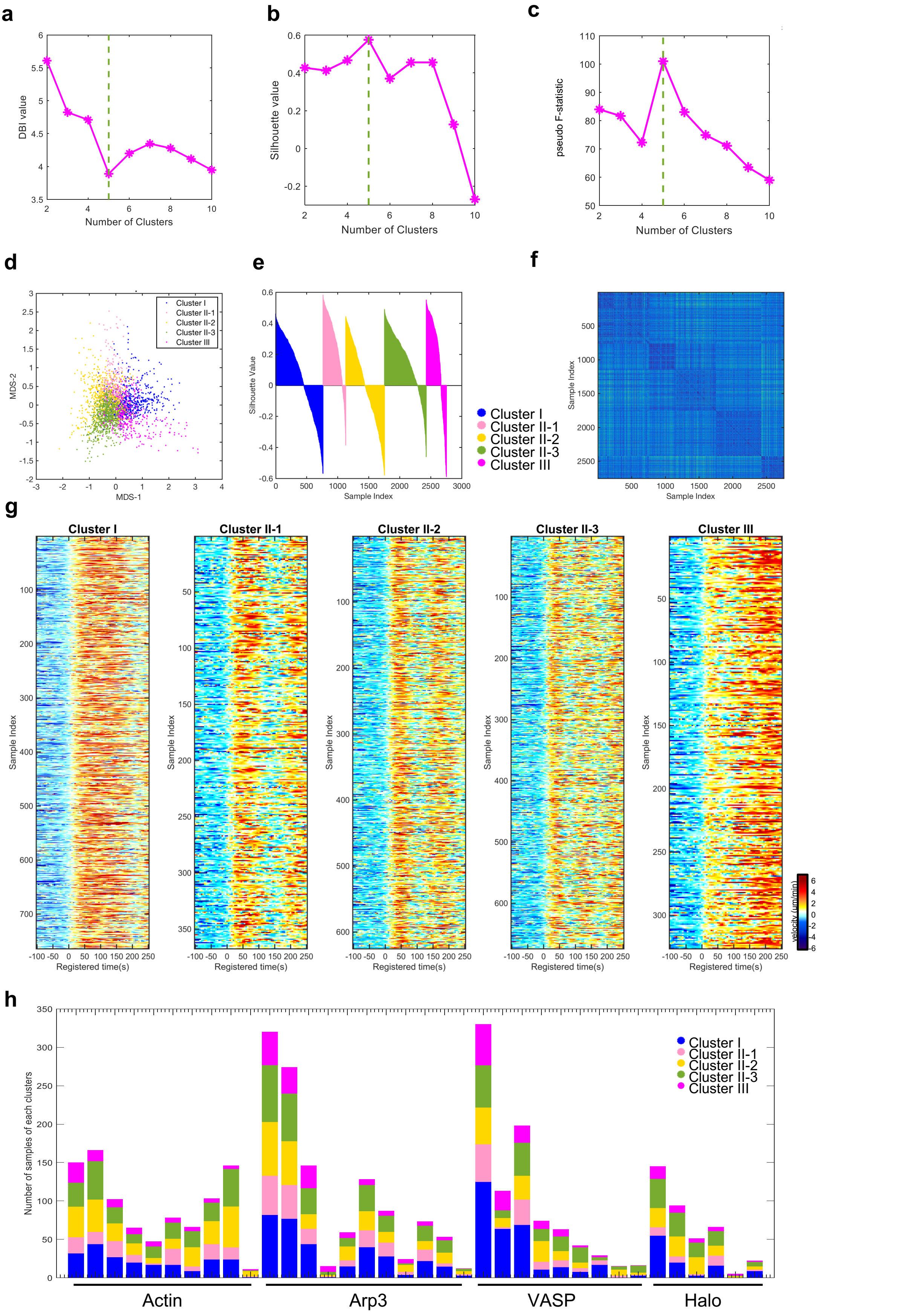

Supplementary Figure 3. Validation of clustering results and sample distribution of clusters (**a**) Davies-Bouldin Index, (**b**) average silhouette, (**c**) Calinski-Harabasz pseudo *F*-statistic as a function of the number of clusters in density peak clustering. **(d)** MDS plot, **(e)** silhouette plot, and **(f)** pair-wise order distance map for the validation of the clustering results. **(g)** Entire raw velocity maps for Cluster I, II-1, II-2, II-3, and III. **(h)** Sample distribution of clusters for individual cells.

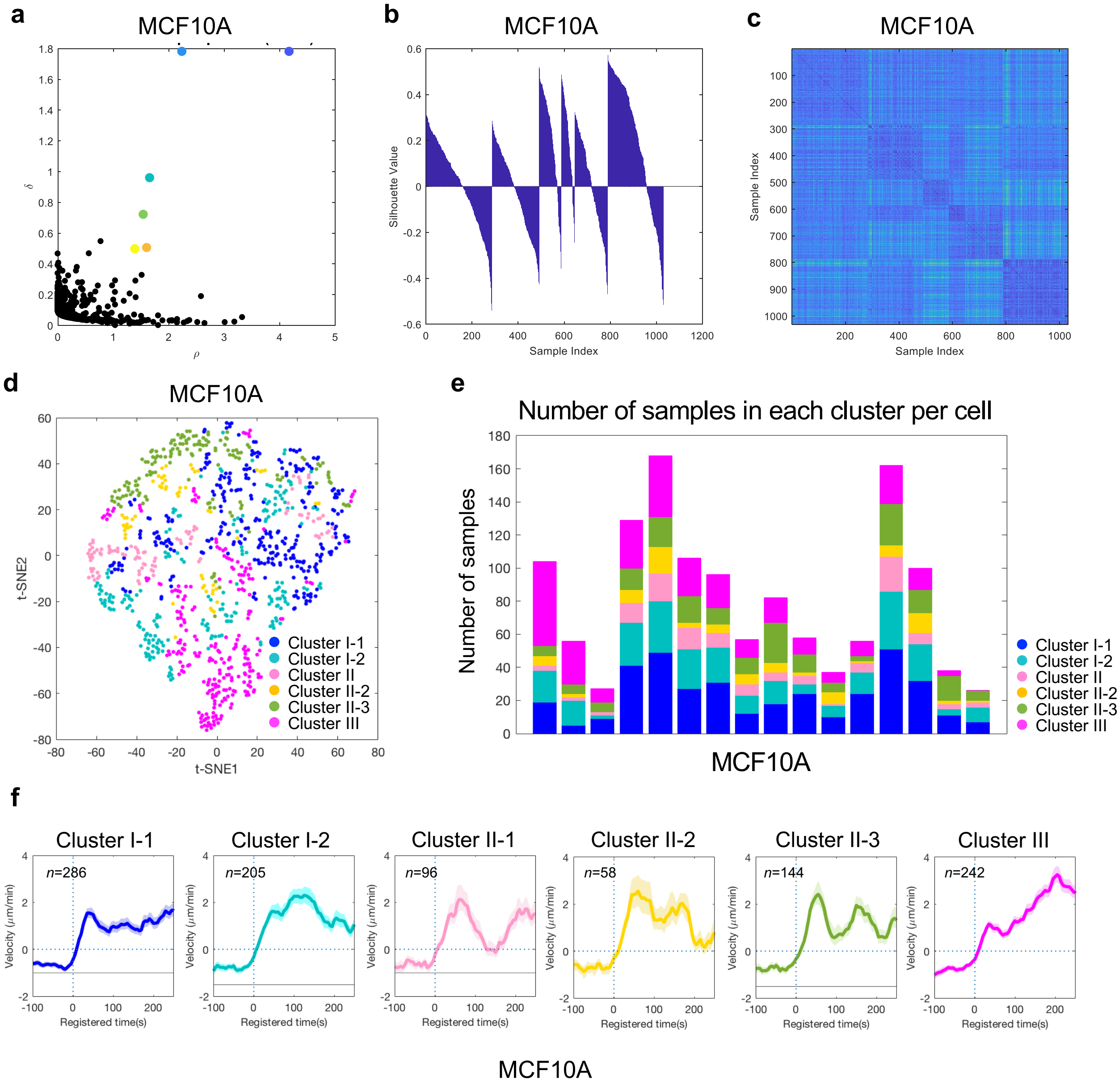

Supplementary Figure 4. Subcellular protrusion phenotypes of MCF10A cells (a) Decision graph of the density peak clustering analysis of protrusion velocities, **(b)** silhouette plot, **(c)** pair-wise distance map, and **(d)** t-SNE plot for the validation of the clustering results. **(e)** Sample distribution of clusters for individual cells. **(f)** Averaged time series of protrusion velocity registered at protrusion onsets (t=0) in each cluster. Solid lines indicate population averages. Shaded error bands indicate 95% confidence intervals computed by bootstrap sampling. *n* is the number of sampled time series. Images from 16 cells were used for the analysis.

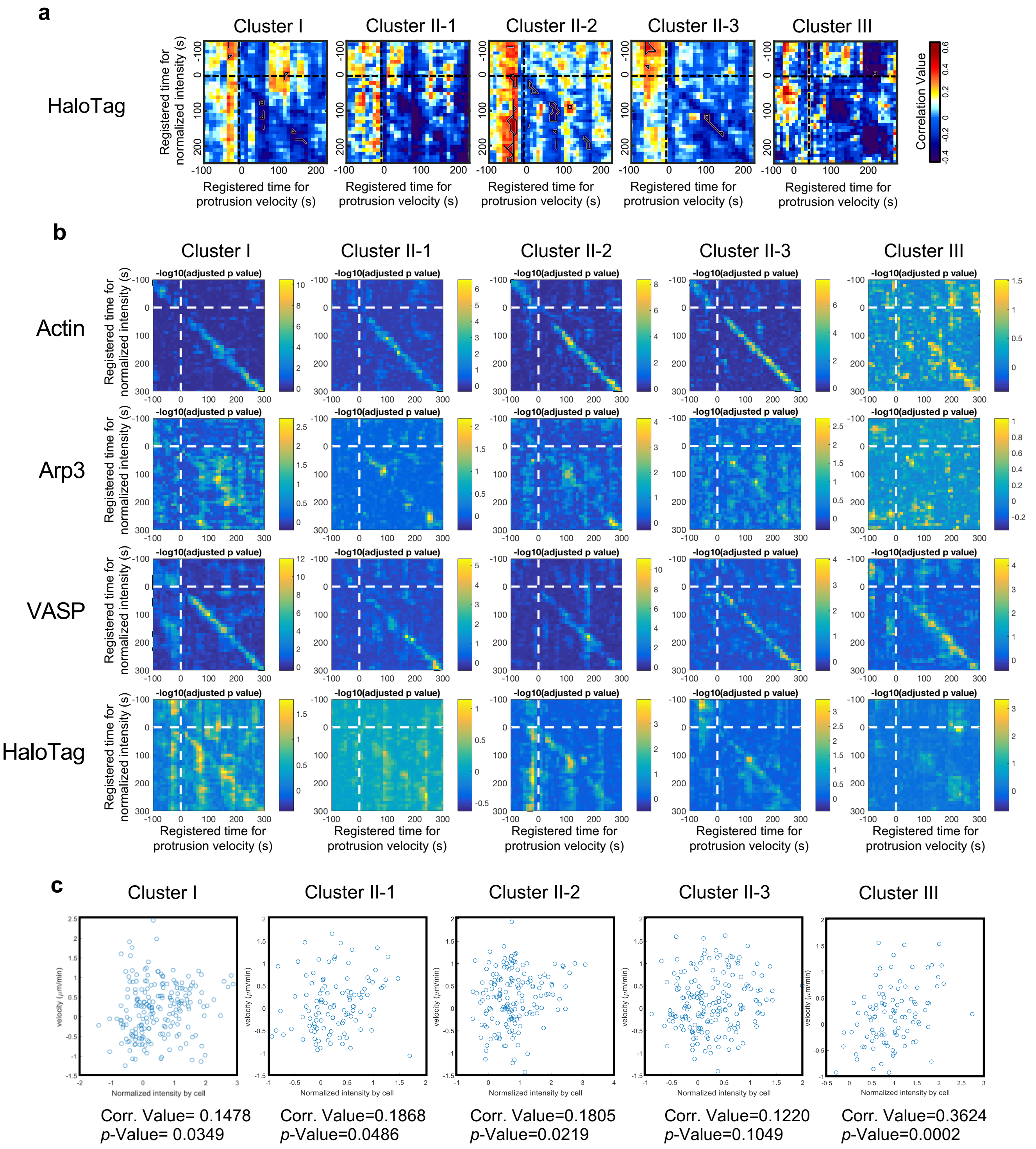

Supplementary Figure 5. Correlation analyses between protrusion velocity and actin regulator dynamics **(a)** Pairwise Pearson's correlation coefficients of protrusion velocity and HaloTag intensity time series registered relative to protrusion onset. The regions surrounded by the black lines are statistically significant correlation by Benjamini-Hochberg multiple hypothesis testing. **(b)** $-\log_{10}$(adjusted *p*-value) of pairwise Pearson's correlation coefficients of protrusion velocity and fluorescence intensity time series. **(c)** Scatter plots and Pearson's correlation coefficients of early Arp3 intensities and late protrusion velocities in each cluster. The permutation *t*-test was used to calculate the *p*-values.

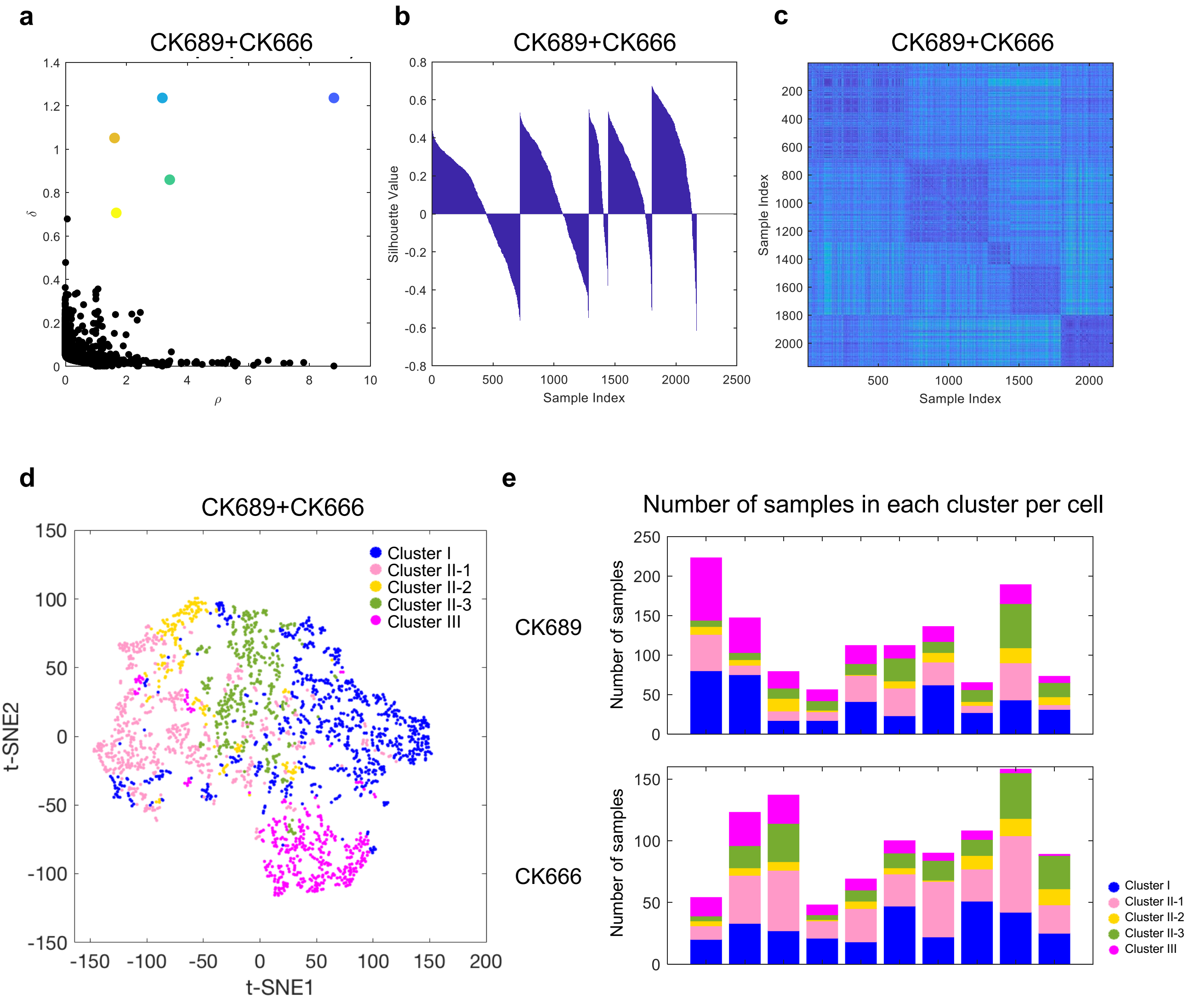

Supplementary Figure 6. Subcellular protrusion phenotypes of PtK1 cells treated with CK689 or CK666 (a) Decision graph of the density peak clustering analysis of protrusion velocities, **(b)** silhouette plot, **(c)** pair-wise distance map, and **(d)** t-SNE plot for the clustering results. **(e)** Sample distribution of the clusters for individual cells treated with CK689/CK666.

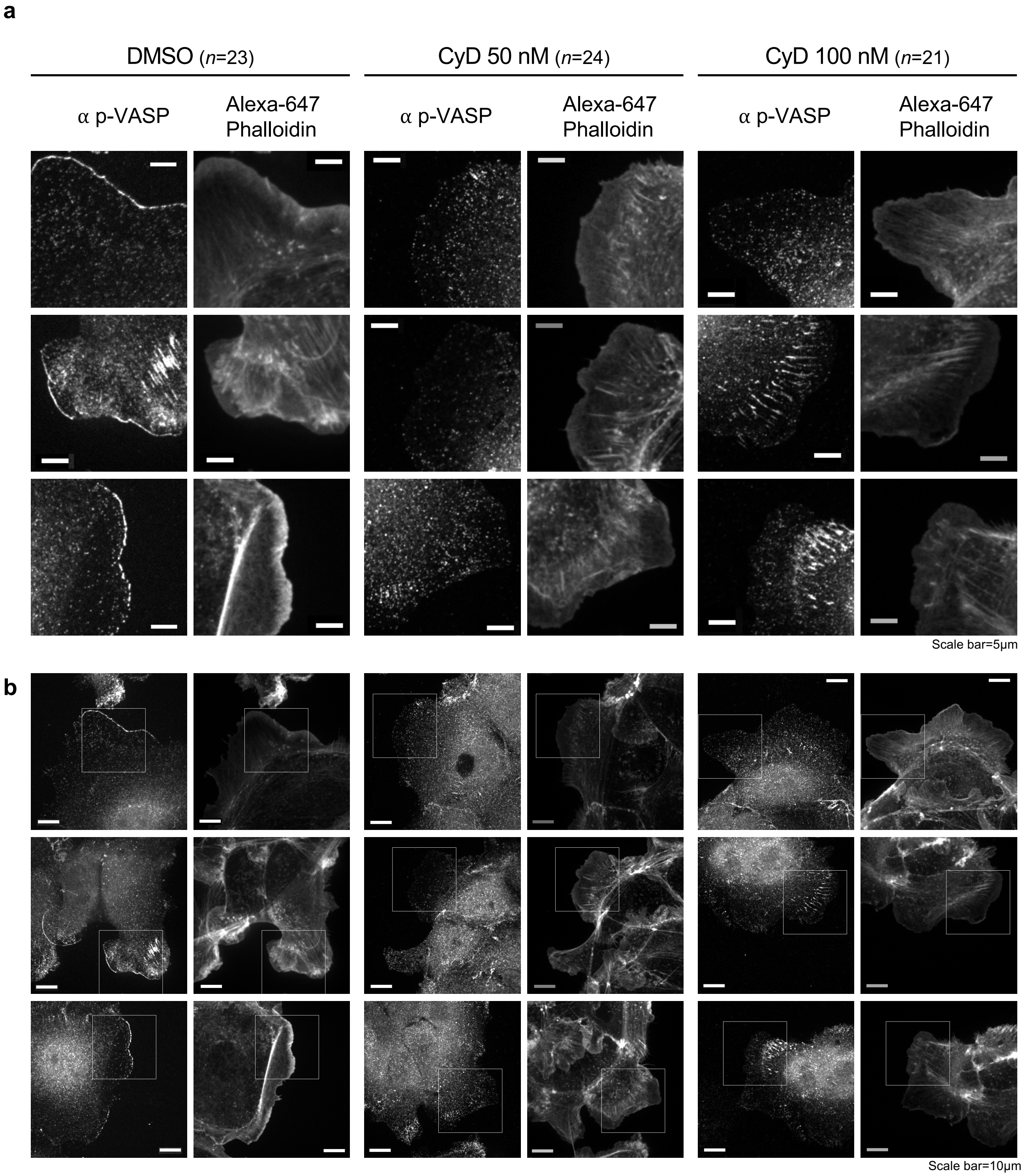

Supplementary Figure 7. Validation of effects of Cytochalasin D on VASP in the lamellipodia of PtK1 cells (a-b) Immunofluorescence images of PtK1 cells stained with α p-VASP and Phalloidin with and without Cytochalasin D treatment. (a) are the magnified images from (b). The number of images taken for DMSO, CyD (50 nM) and CyD (100 nM)-treated cells are 23, 24 and 21 respectively. Representative 3 images are selected and presented.

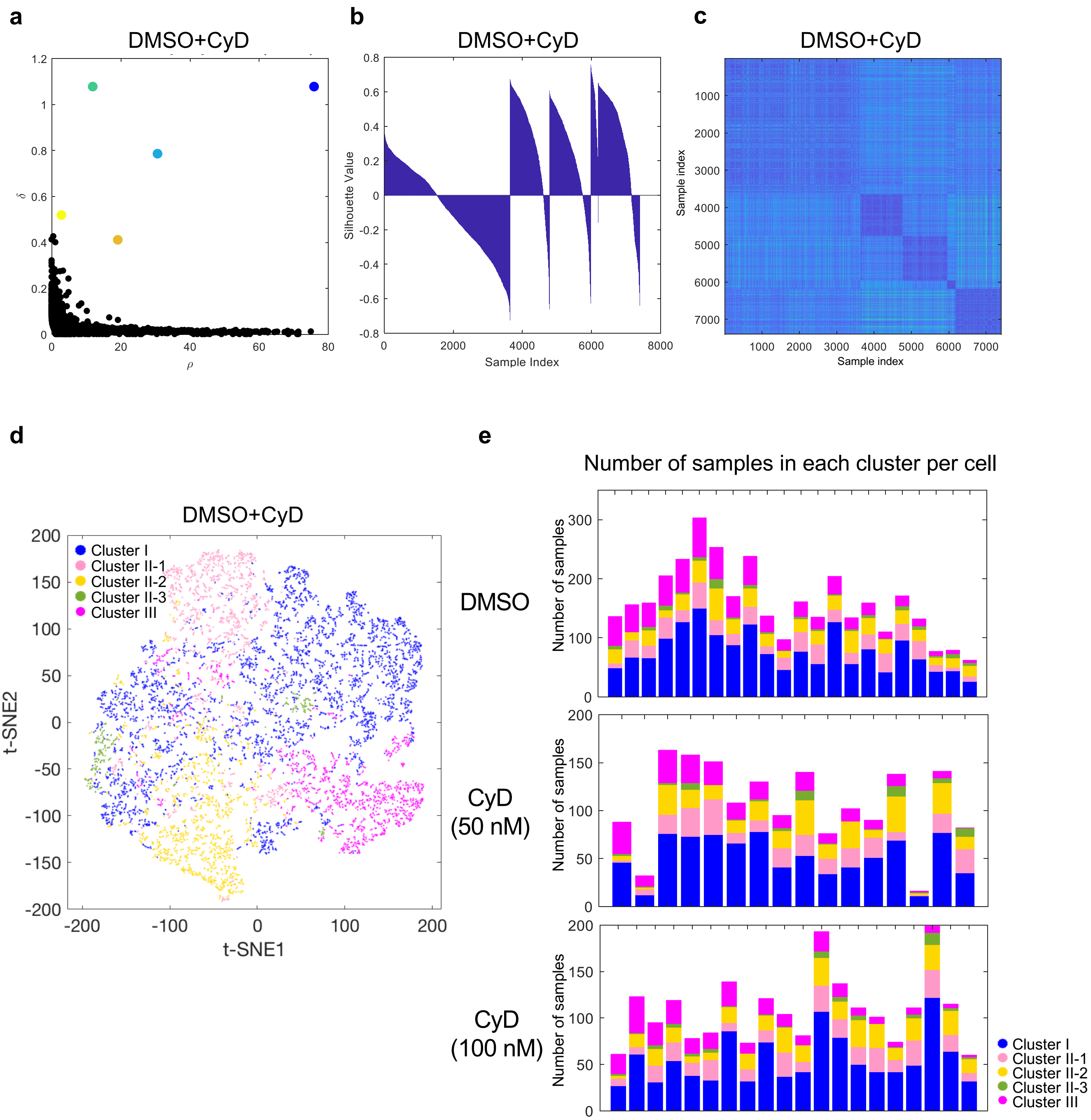

Supplementary Figure 8. Subcellular protrusion phenotypes of PtK1 cells treated with Cytochalasin D (a) Decision graph of the density peak clustering analysis of protrusion velocities, **(b)** silhouette plot, **(c)** pair-wise distance map, and **(d)** t-SNE plot for the clustering result. **(e)** Sample distribution of the clusters for individual cells treated with DMSO and Cytochalasin D.

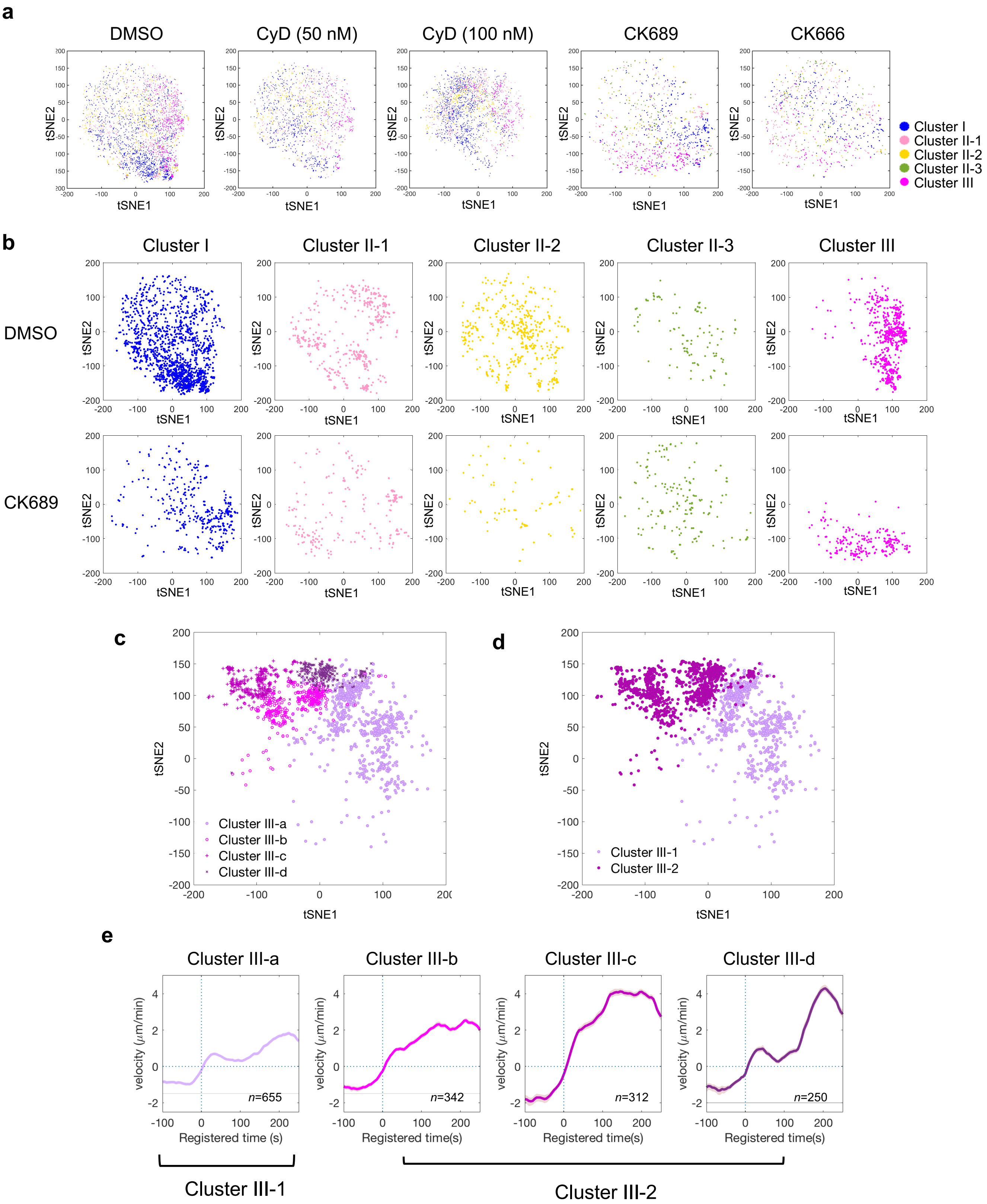

Supplementary Figure 9. Sub-clustering of accelerating protrusion (a) t-SNE plots of denoised protrusion velocity time series overlaid with the cluster assignments. **(b)** t-SNE plots of denoised protrusion velocity time series of individual clusters in control (DMSO/CK689) cells. **(c)** Sub-clustering of Cluster III by community detection. **(d)** Final sub-clustering by merging Cluster III-b/c/d to Cluster III-2. **(e)** Average time series of protrusion velocity registered at protrusion onsets (t=0) in each sub-clusters before merging. Solid lines indicate population averages. Shaded error bands indicate 95% confidence intervals computed by bootstrap sampling.

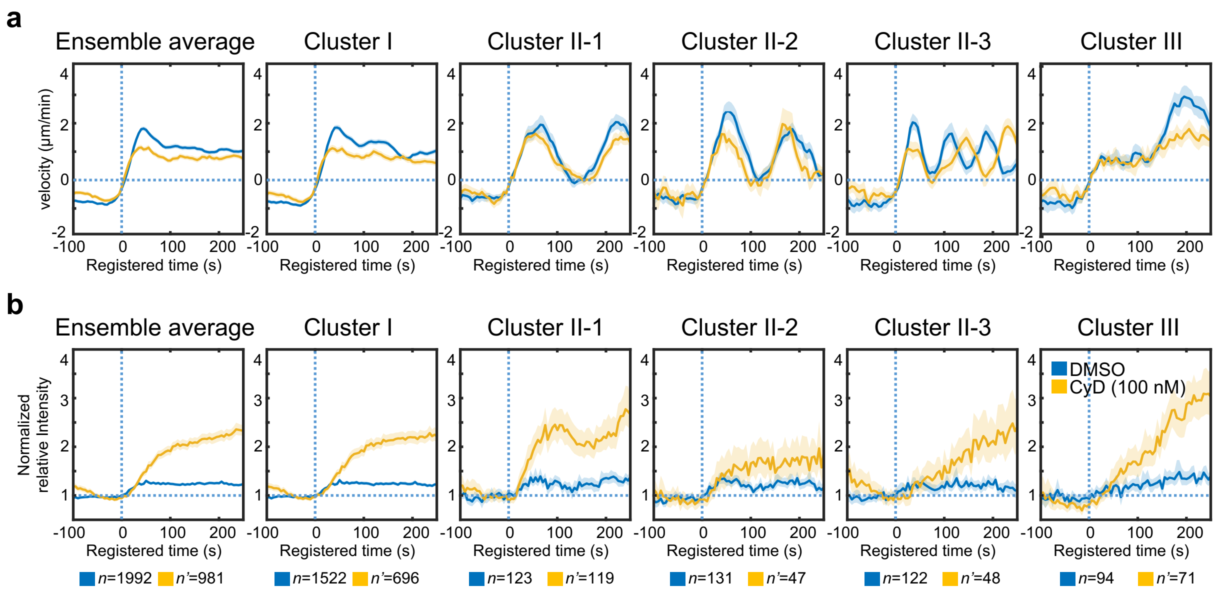

Supplementary Figure 10. Changes in Arp3 recruitment upon Cytochalasin D treatment (a) Ensemble averaged velocity time series of entire samples and averaged velocity time series of each cluster upon DMSO or Cytochalasin D (100 nM) treatment in Arp3-expressing cells. **(b)** Ensemble averaged normalized fluorescence intensity series of entire samples and averaged normalized fluorescence intensity time series in each cluster upon DMSO or Cytochalasin D treatment in Arp3-expressing cells. All time series are registered with respect to protrusion onset (t=0). Solid lines indicate population averages. Shaded error bands indicate 95% confidence intervals computed by bootstrap sampling. *n* is the number of time-series used for the analysis for DMSO treated GFP-Arp3 expressing cells, and *n'* is the number of time-series used for the analysis for CyD treated GFP-Arp3 expressing cells. The numbers of the cells imaged for DMSO and CyD treatment are 15 and 11 respectively.

SUPPLEMENTARY NOTES

Supplementary Note 1: Evaluation of the role of each component of time series clustering

Our time series clustering primarily consisted of SAX (dimensional reduction), ACF distance (dissimilarity measure with Autocorrelation Function) and Density Peak clustering. To identify the role of each component in our pipeline, we systematically tested the performance of the clustering results by replacing each component with other methods. First, without SAX for dimensional reduction, two different distance dissimilarity measures, ACF and Euclidean distances were tested. When Euclidean distance was applied without SAX, we could not find distinct cluster structure using the Density Peak clustering (Supplementary Fig. 1a), and we were not able to extract periodic and accelerating protrusions from the de-noised velocity samples (Supplementary Fig. 1a-c). When we applied community detection using Euclidean distance without SAX, we still could not identify periodic and accelerated protrusions (Supplementary Fig. 1d-f). When the ACF distance was used without SAX, we were able to identify accelerating protrusions (Supplementary Fig. 1g-j) using Density Peak clustering, but we could not find the full spectrum of the periodic protrusion patterns (Supplementary Fig. 1j). However, we were able to identify periodic and accelerating protrusion using Community Detection method, when the ACF distance was used without SAX (Supplementary Fig. 1k-m). Furthermore, when we tested the clustering using SAX and its Euclidean distance (the lower bound of Euclidean distance in SAX), we were not able to identify distinct temporal patterns of protrusion velocity (Supplementary Fig. 1n-q). Taken together, we concluded that the ACF distance is the most important factor which allowed us to extract the periodic and accelerating protrusion.

When we used ACF distance with SAX, the Community Detection method was able to identify both accelerating protrusion and three periodic protrusions when the number of clusters was set to be six (Supplementary Fig. 2 h-i). However, it could not identify one periodic pattern when the number of clusters is set to be five (Supplementary Fig. 2 f-g). Since the silhouette values in Supplementary Fig. 2f,h is larger than those in Supplementary Fig. 1k, SAX seems to play a role in making cluster tighter and cleaner by

reducing the local data fluctuation. Using ACF distance with SAX, density Peaks can extract both acceleration protrusion and three periodic protrusions (Fig. 2).

Finally, we also applied the conventional K-means clustering algorithm to our data using ACF distance with SAX (Supplementary Fig. 2a-e). First, we determine the optimal number of clusters to be seven using both Davies–Bouldin Index and Silhouette criteria (Supplementary Fig. 2a). Due to the random initialization of cluster centers, we showed two clustering results from K-means (Supplementary Fig. 2b-c and Supplementary Fig. 2d-e), where both acceleration protrusion and periodic protrusions were identified. Therefore, the combination of ACF and SAX, three clustering methods (K-means, Density Peaks, and Community Detection) were able to isolate the acceleration protrusion and three periodic protrusions patterns. However, in K-means and Community Detection, the optimal number of clusters is not easily determined. To delineate both periodic and accelerating protrusions, we needed to use the larger number of clusters, which resulted in small numbers of samples in each cluster. Density Peaks not only provided the information to determine the number of clusters but also effectively discovers both periodic and accelerating protrusions with a relatively smaller number of clusters. Moreover, the density peak is a deterministic method while K-means and community detection are involved in the random initiation and random walk. Therefore, the Density Peak can produce more reproducible results. These results were summarized in Supplementary Table 2.

Supplementary Note 2: The correlation between early Arp2/3 and late protrusion velocities

The significant and strong instantaneous correlation between VASP and the protrusion velocity begins to appear 100 seconds after protrusion onset (Fig. 4h, Cluster III), along with no correlation between actin and the protrusion velocity in Cluster III (Fig. 4f, Cluster III). Since the Arp3 intensity in Cluster III reached the maximum at approximately this time point (Fig. 3c, Cluster III), we hypothesized that Arp2/3 in the early phase of Cluster III might play an important role. To test this idea, we integrated early Arp3 intensities between 0 to 50 seconds and correlated them with average protrusion velocities between 150 and 200

seconds in each cluster (Fig. 4i, Supplementary Fig. 5c). The Pearson's correlation coefficient in Cluster III was significant (0.36, p = 0.0002, permutation t-test) whereas those in other clusters were not or marginally significant (Supplementary Fig. 5c). This is consistent with our hypothesis that Arp2/3 may be important in initiating cell protrusion in Cluster III.

Supplementary Note 3: Prediction of accelerating protrusion by VASP dynamics

Since VASP intensities were well correlated with protrusion velocities, we investigated whether VASP intensities contain sufficient information to predict protrusion phenotypes. To this end, we applied supervised learning approaches to further validate that VASP intensity time series can predict the acceleration phenotype. Using support vector machine (SVM), deep neural network (DNN), and random forest (RF), we built classifiers from the normalized intensities of actin, Arp3, and VASP to distinguish the non-accelerating (Clusters I and II-1/2/3) and accelerating (Cluster III) protrusion phenotypes. As a result, classifiers that were trained using VASP intensities could distinguish accelerating protrusions from non-accelerating protrusions with a significantly higher accuracy (Fig. 4j) and MCC (Matthews correlation coefficient) (Fig. 4k) than the classifiers trained using the actin and Arp3 intensities (p-values in Supplementary Table 4, two-tailed K-S test). Despite the modest classification accuracy, this difference suggests not only that VASP correlated with protrusion velocity but also that the correlation is strong enough to predict the accelerating protrusion phenotype.
