# Supplementary material for: Deconvolution of subcellular protrusion heterogeneity and the underlying actin regulator dynamics from live cell imaging
Source: Nat Commun. 2018 Apr 27;9:1688. doi: 10.1038/s41467-018-04030-0 (PMC5923236; doi:10.1038/s41467-018-04030-0)
Supplement: Supplementary file 2 — Description of Additional Supplementary Files [file 41467_2018_4030_MOESM2_ESM.pdf]

Description of Additional Supplementary Files

File Name: Supplementary Movie 1
Description: **Representative cluster-assigned movie of a PtK1 cell stained with CellMask DeepRed.** Blue: Cluster I, Light Pink: Cluster II-1, Yellow: Cluster II-2, Green: Cluster II-3, Magenta: Cluster III. Scale bar: 20 µm. Original movie captured by spinning disc confocal microscopy at a 5s frame interval; replay at 30 frames per second.

File Name: Supplementary Movie 2
Description: **Representative cluster-assigned movie of a PtK1 cell stained with CellMask DeepRed treated with 50 µM CK689.** Blue: Cluster I, Cyan: Cluster II-1, Yellow: Cluster II-2, Red: Cluster II-3, Magenta: Cluster III. Scale bar: 20 µm. The original movie captured by spinning disc confocal microscopy at a 5s frame interval; replay at 30 frames per second.

File Name: Supplementary Movie 3
Description: **Representative cluster-assigned movie of a PtK1 cell stained with CellMask DeepRed treated with 50 µM CK666.** Blue: Cluster I, Cyan: Cluster II-1, Yellow: Cluster II-2, Red: Cluster II-3, Magenta: Cluster III. Scale bar: 20 µm. The original movie captured by spinning disc confocal microscopy at a 5s frame interval; replay at 30 frames per second.

File Name: Supplementary Movie 4
Description: **Representative cluster-assigned movie of a PtK1 cell stained with CellMask DeepRed treated with DMSO.** Blue: Cluster I, Cyan: Cluster II-1, Yellow: Cluster II-2, Red: Cluster II-3, Magenta: Cluster III. Scale bar: 20 µm. The original movie captured by spinning disc confocal microscopy at a 5s frame interval; replay at 30 frames per second.

File Name: Supplementary Movie 5
Description: **Representative cluster-assigned movie of a PtK1 cell stained with CellMask DeepRed treated with 50 nM Cytochalasin D.** Blue: Cluster I, Cyan: Cluster II-1, Yellow: Cluster II-2, Red: Cluster II-3, Magenta: Cluster III. Scale bar: 20 µm. The original movie captured by spinning disc confocal microscopy at a 5s frame interval; replay at 30 frames per second.

File Name: Supplementary Movie 6
Description: **Representative cluster-assigned movie of a PtK1 cell stained with CellMask DeepRed treated with 100 nM Cytochalasin D.** Blue: Cluster I, Cyan: Cluster II-1, Yellow: Cluster II-2, Red: Cluster II-3, Magenta: Cluster III. Scale bar: 20 µm. The original movie captured by spinning disc confocal microscopy at a 5s frame interval; replay at 30 frames per second.
